# Single cell and bulk transcriptome analysis identified oxidative stress response-related features of Hepatocellular Carcinoma

**DOI:** 10.3389/fcell.2023.1191074

**Published:** 2023-09-28

**Authors:** Shuqiao Zhang, Xinyu Li, Yilu Zheng, Jiahui Liu, Hao Hu, Shijun Zhang, Weihong Kuang

**Affiliations:** ^1^ First Affiliated Hospital of Guangzhou University of Chinese Medicine, Guangzhou University of Chinese Medicine, Guangzhou, Guangdong, China; ^2^ Department of Hematology, The Seventh Affiliated Hospital, Sun Yat-sen University, Guangzhou, Guangdong, China; ^3^ Department of Traditional Chinese Medicine, The First Affiliated Hospital, Sun Yat-sen University, Guangzhou, Guangdong, China; ^4^ Guangdong Key Laboratory for Research and Development of Natural Drugs, Dongguan Key Laboratory of Chronic Inflammatory Diseases, School of Pharmacy, The First Dongguan Affiliated Hospital of Guangdong Medical University, Guangdong Medical University, Dongguan, Guangdong, China

**Keywords:** oxidative stress, Hepatocellular carcinoma, single-cell transcriptome, immune microenvironment, prognosis

## Abstract

**Background:** Hepatocellular Carcinoma (HCC) is a common lethal digestive system tumor. The oxidative stress mechanism is crucial in the HCC genesis and progression.

**Methods:** Our study analyzed single-cell and bulk sequencing data to compare the microenvironment of non-tumor liver tissues and HCC tissues. Through these analyses, we aimed to investigate the effect of oxidative stress on cells in the HCC microenvironment and identify critical oxidative stress response-related genes that impact the survival of HCC patients.

**Results:** Our results showed increased oxidative stress in HCC tissue compared to non-tumor tissue. Immune cells in the HCC microenvironment exhibited higher oxidative detoxification capacity, and oxidative stress-induced cell death of dendritic cells was attenuated. HCC cells demonstrated enhanced communication with immune cells through the MIF pathway in a highly oxidative hepatoma microenvironment. Meanwhile, using machine learning and Cox regression screening, we identified PRDX1 as a predictor of early occurrence and prognosis in patients with HCC. The expression level of PRDX1 in HCC was related to dysregulated ribosome biogenesis and positively correlated with the expression of immunological checkpoints (PDCD1LG2, CTLA4, TIGIT, LAIR1). High PRDX1 expression in HCC patients correlated with better sensitivity to immunotherapy agents such as sorafenib, IGF-1R inhibitor, and JAK inhibitor.

**Conclusion:** In conclusion, our study unveiled variations in oxidative stress levels between non-tumor liver and HCC tissues. And we identified oxidative stress gene markers associated with hepatocarcinogenesis development, offering novel insights into the oxidative stress response mechanism in HCC.

## 1 Introduction

Hepatocellular Carcinoma (HCC), the most prevalent malignant tumor of the digestive system, is the world’s third cause of mortality from cancer (Ganesan and Kulik, 2023). Chronic alcohol consumption, diabetes or obesity, metabolic syndromes, and infection by Hepatitis B virus are key factors responsible for HCC progression, which promotes cirrhosis, ultimately HCC (Mittal and El-Serag, 2013; Kim and Viatour, 2020; Zhang et al., 2023). Now, therapeutic options for HCC are evolving from surgical treatments such as liver transplantation, surgical resection, percutaneous ablation, and radiotherapy to diversified immunotherapeutic strategies (Vogel et al., 2022). However, the efficacy of immunotherapy for HCC still needs to be improved and faces significant challenges. It has an association with the microenvironment of HCC.

The HCC microenvironment is mainly composed of cellular components such as tumor cells, tumor-infiltrating lymphocytes, and tumor-associated neutrophils, as well as noncellular components such as cytokines and chemokines (Anderson and Simon, 2020). The microenvironment of HCC differs greatly from that of normal liver tissues, in which inflammation is one of the leading environmental cues that mediate tumorigenesis. It has been demonstrated that acute and chronic inflammation can induce oxidative stress in the liver. During this process, hepatocytes become affected by reactive oxygen species (ROS) generated from electron leakage from mitochondrial electron transport, leading to the activation of oncogenic pathways (Takaki and Yamamoto, 2015). The interactions among the HCC microenvironment are also rather complex. ROS in the environment can promote malignant transformation via excessive activation of cell growth, differentiation, and survival signaling pathways such as PI3K/Akt/mTOR, and MAPK/ERK (Aboelella et al., 2021).

Oxidative stress is a circumstance that occurs when there is an imbalance between the oxidative and antioxidant effects within tissues (Forman and Zhang, 2021). It is not only one of the major causes of hepatic inflammatory diseases but intimately associated with the generation and progression of HCC (Sasaki, 2006). Our study analyzed the effect of oxidative stress on cells in the microenvironment by combining single-cell and bulk sequencing data from the non-tumor liver and HCC tissues and identified oxidative stress-related genes that have a critical impact on the survival of HCC patients.

## 2 Materials and methods

### 2.1 Data acquisition

Raw single-cell transcriptome profiling data for ten HCC patients from two relevant sites, primary tumor (HCC01T, HCC02T, HCC03T, HCC04T, HCC05T, HCC06T, HCC07T, HCC08T, HCC09T, and HCC10T) and adjacent non-tumor liver (HCC03N, HCC04N, HCC05N, HCC06N, HCC07N, HCC08N, HCC09N, and HCC10N), was achieved from GSE149614 dataset in Gene Expression Omnibus (GEO) database (https://www.ncbi.nlm.nih.gov/geo/). Bulk RNA-seq data of HCC patients were collected as follows, TCGA-LIHC dataset from The Cancer Genome Atlas (https://portal.gdc.cancer.gov/repository) database, HCCDB18 dataset from Integrative Molecular Database of Hepatocellular Carcinoma (http://lifeome.net/database/hccdb/download.html) database, and GSE datasets (GSE76427, GSE54236, GSE36376, GSE69715, GSE121248, GSE107170, and GSE45267) from GEO database. 110 normal liver samples RNA-seq data were retrieved from Genotype-Tissue Expression (GTEx, https://gtexportal.org/home/) database. The samples information in all data sets can be seen in Supplementary Table S1.

### 2.2 Data processing

We used “Seurat” packages (Hao et al., 2021) in R software to process the single cell data. Gene number, relative hemoglobin, and mitochondrial and ribosomal abundance (Supplementary Figure S1), indicating that the cellular readouts were comparable between samples and no transcriptional batch effects were observed. Cells with <1,500 or >20,000 detected genes containing mitochondrial genome >5% were excluded. Indicating that the cellular readouts were comparable between samples and no transcriptional batch effects were observed. Cells with <2,500 or >20,000 detected genes containing mitochondrial genome >4% were excluded. Next, single-cell data were normalized, and variable genes were hunted by the “SCTransform” method. After, 20 most powerful principal components were found by PCA analysis (Supplementary Figure S2). Further dimension reduction of those principal components was proceeded by the UMAP method to visualize cell distribution. Cell types of principal components were annotated by “CellMarker” (http://xteam.xbio.top/CellMarker/) and “PanglaoDB” (https://panglaodb.se/) databases. Cell-cell communication were conducted by “CellChat” R package. In addition, Bulk RNA-sequencing expression datasets were normalized by log2 (FPKM+1) through “limma” package in R.

### 2.3 Pathway analysis

The verified pathways containing gene sets provided in the Molecular Signatures database were utilized by Gene Set Variation Analysis (GSEA) (Mo et al., 2003) to analyze the biological functions of single-cell data. Gene-related differential expression biological processes in transcriptome data were analyzed by single-sample Gene Set Variation Analysis (ssGSEA) (Rooney et al., 2015; Zhang et al., 2021b). Metascape (a gene annotation and analysis resource, https://metascape.org/) and molecular complex detection (MCODE) (Osterman et al., 2020) methods analyzed oxidative stress-related genes’ interactions and functional pathways.

### 2.4 Analysis of cell–cell communications

CellChat (Jin et al., 2021) toolkit in R enables the analysis and interpretation of cell-cell communication within complex biological systems. Using CellChat, the gene expression profiles are analyzed to identify ligand-receptor pairs between different cell types. CellChat provides a comprehensive database of known ligand-receptor interactions, which is used to identify potential communication channels between cell types. It evaluates the significance of ligand-receptor pairs in cell-cell interactions using correlation analysis (Cowen et al., 2017; Ronen and Akalin, 2018; Szklarczyk et al., 2019). Once the ligand-receptor pairs are identified, CellChat analyzes the signaling pathways associated with these interactions. We utilize information from the secreted signaling pathways database (Jin et al., 2020) in CellChat to determine the ligand-receptor interactions in intercellular communication.

### 2.5 Screening and evaluation of oxidative stress response-related gene markers with diagnostic value by multiple machine learning algorithms

Due to the relatively small number of non-tumor liver tissue samples in TCGA-LIHC dataset compared to HCC samples, we used “Sva” package to merge the normalized GTEX dataset with the TCGA dataset and remove batch effects. The two datasets after removal of batch effects are shown in Supplementary Figure S3. Model building was based on the oxidative stress-related genes expression profile of merged dataset. The samples in the merged dataset were randomly split into training sets and test sets in the proportion 7:3, respectively. First, we applied machine learning algorithms, including extreme gradient boosting (XGB) (Li et al., 2023), support vector machine (SVM) (Huang et al., 2023), generalized linear model (GLM) (Wang et al., 2023), and random forest (RF) (Su et al., 2023), to create models that screened for significant oxidative stress-related gene feature variables. All machine learning methods used default settings to generate models. The grid search function in the “caret” R package was used to modify model parameters automatically, which were tested using 5-fold cross-validation. Secondly, we interpreted the four machine learning models obtained above using the explaining features function of the “Dalex” R package. The explaining features, including model performance analysis and variable importance analysis, were then used to analyze the four models described above. Model performance analysis visualized the performance distribution of the four models. The models’ cumulative residual and box plot distribution diagrams were drawn, respectively. The variable importance analyzes the relative importance of different variables in the model to the model’s prediction. It explains the impact of missing the variable on the prediction value of the response variable through the root mean square error loss function. Thirdly, the area under the curve was obtained by receiver operating characteristic curves (ROC) analysis to identify the best machine-learning model. The top six significant variable features from the optimal model were considered diagnostic gene markers for predicting the occurrence of HCC. Finally, for clinical usability, we used these diagnostic gene markers to conduct nomogram model construction and to judge their prediction error from the actual situation through the calibration curves. Additionally, the prediction performance of the diagnostic gene markers was examined by seven external independent cohorts, including GSE76427, GSE54236, GSE36376, GSE69715, GSE121248, GSE107170, and GSE45267.

### 2.6 Prognostic value analysis of PRDX1 in HCC

We further analyzed six oxidative stress-related gene markers (GPX4, HMOX1, PRDX1, FOS, PRDX5, and TXN) with the diagnostic value, which from the machine learning analysis steps. To judge whether they also play a role in predicting the prognosis of HCC patients. Univariate and multivariate Cox regression analyses were performed for these six genes based on survival and gene expression information of HCC patients in the TCGA cohort. After screening, PRDX1 was correlated with the prognostic survival status of HCC patients. The patients with HCC were then classified into high and low-expression groups by median cut-off value, using Kaplan-Meier survival estimate (KM) and time-dependent ROC analysis to assess whether PRDX1 was a prognostic factor. The survival analysis results were finally validated using an independent HCCDB18 cohort.

### 2.7 Correlation analysis between PRDX1 and immune microenvironment of HCC

To explore the correlation of PRDX1 with the immune microenvironment in HCC. We used six algorithms [CIBERSORT (Chen et al., 2018), CIBERSOR-ABS (Wang et al., 2020), QUANTISEQ (Plattner et al., 2020), MCP-counter (Zhang et al., 2021a), XCELL (Aran et al., 2017), and TIMER (Li et al., 2017)] for immune cell infiltration analyses to assess HCC individuals with the different expression level of PRDX1. Differences in immune function between different PRDX1 expression groups were then analyzed using the “GSVA” package.

### 2.8 Statistical analysis

**FIGURE 1:**
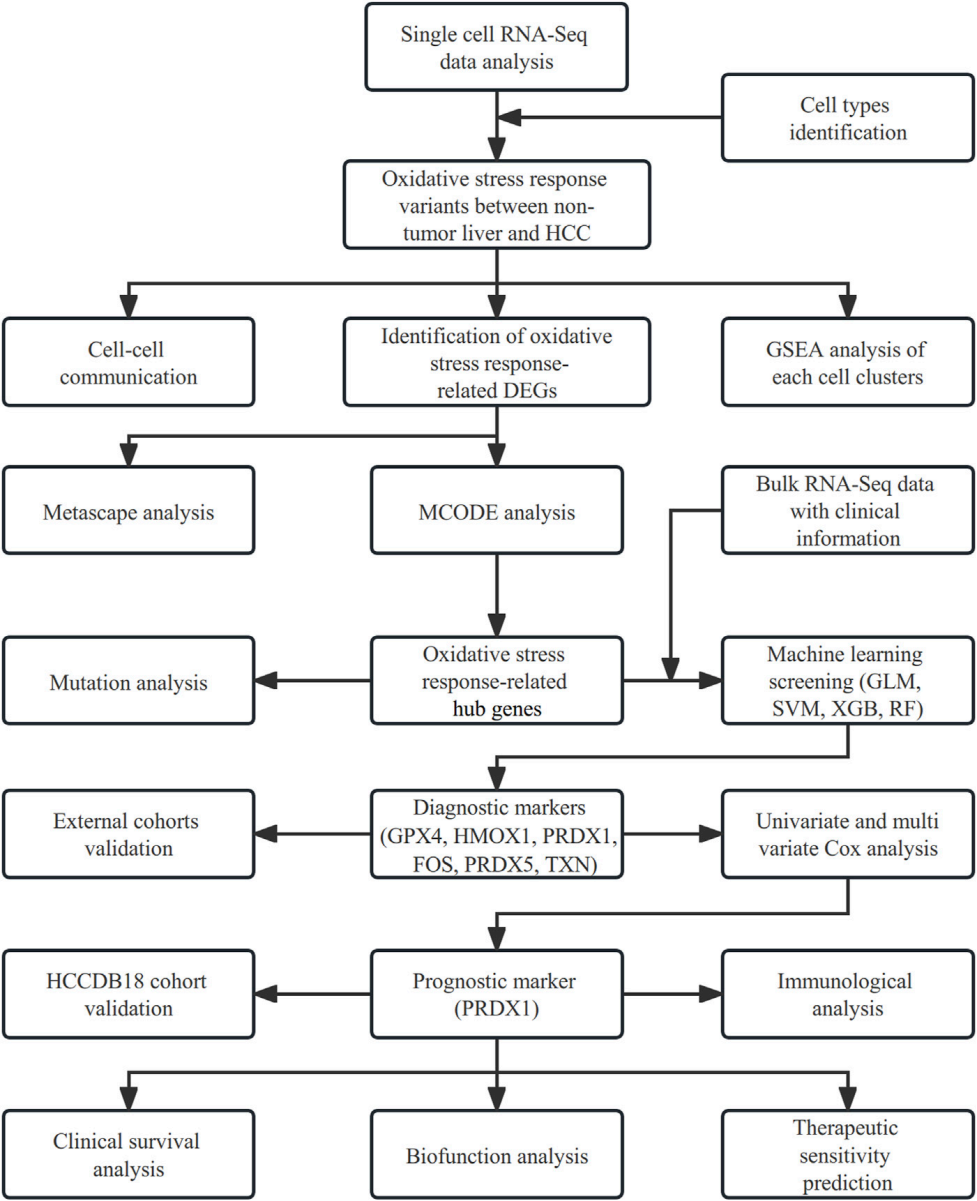
Flow chart of our study.

The analyses of our study were all employed by R 4.1.1 software. Analyses used a false discovery rate (FDR) < 0.05 from the *p*-value, which was adjusted by the Bonferroni method. Figure 1 displays a flow chart outlining our study.

## 3 Results

### 3.1 Landscape of the cell composition in non-tumor and HCC tissues

**FIGURE 2:**
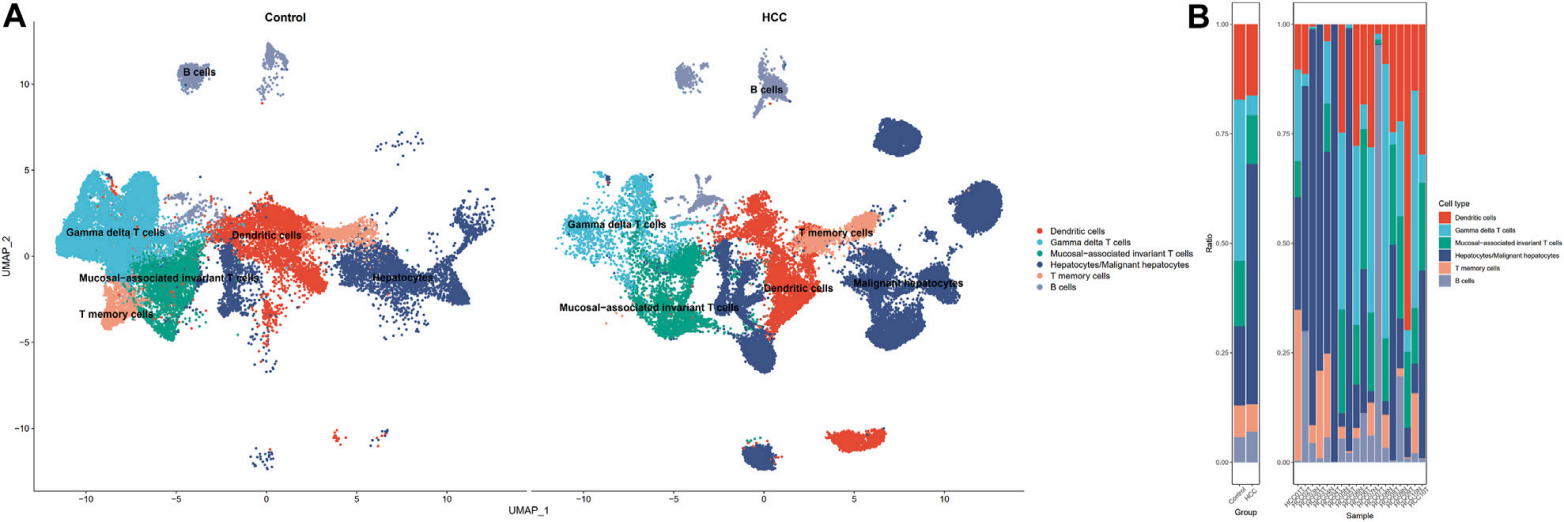
Single-cell profiling of HCC and adjacent non-tumor samples. **(A)** UMAP distribution of cell types in HCC *versus* non-tumor (control) tissues. **(B)** Contrasting differences in cell ratios in the two tissues.

The GSE149614 dataset was utilized for single-cell analysis in our study. After quality control for scRNA-seq data, we filtered out 28,687 cells in tumor-adjacent noncancerous tissues (control) and 34,414 cells in HCC tissues for further study. We characterized the cellular landscape using cell classification marker genes identified in previous studies (Ronen and Akalin, 2018) (Supplementary Figure S4). Following, seven cell types, including dendritic cells, gamma delta T cells, mucosal−associated invariant T cells, T memory cells, B cells, and hepatocytes or malignant hepatocyte cells, were identified from the landscape (Figure 2A). When we compared cell type proportions (Figure 2B) between non-tumor liver tissues and HCC tissues, we discovered that the ratio of mucosal-associated invariant T cell, T memory cell, and gamma delta T cell in HCC tissues were significantly decreased. In contrast, the proportion of B cells increased in HCC tissues.

### 3.2 Oxidative stress of cell clusters between non-tumor and HCC samples

**FIGURE 3:**
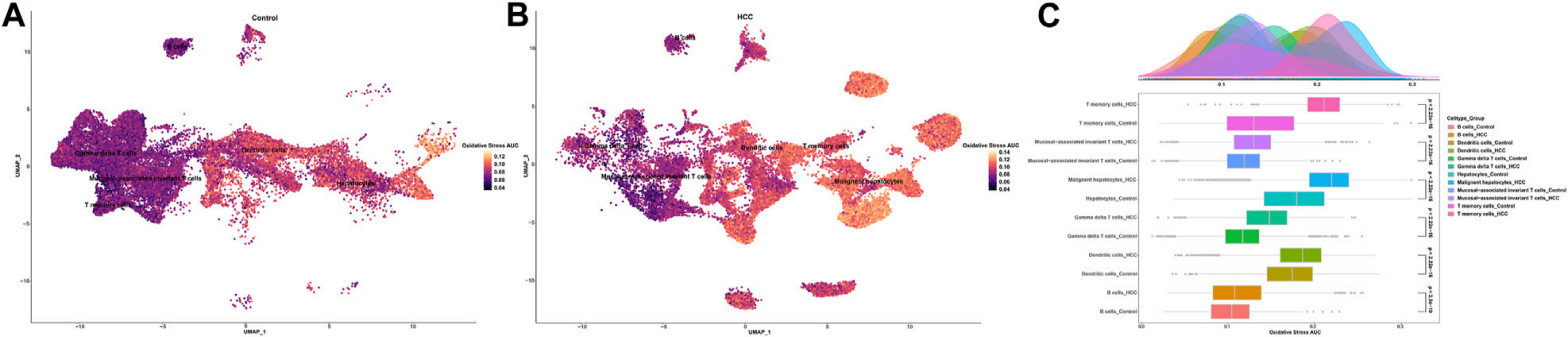
Oxidative stress AUC of cell clusters between the HCC and non-tumor tissues. **(A)** UMAP plot of oxidative stress AUC in non-tumor tissues. **(B)** UMAP plot of oxidative stress AUC in HCC tissues. **(C)** Comparison of oxidative stress AUC of cell clusters between non-tumor tissues and HCC tissues.

To investigate the oxidative stress characteristics of non-malignant samples and HCC samples, oxidative stress AUC (oxidative stress scoring for each cell) was generated based on the expression profile of genes in oxidative stress response collected from the Molecular Signatures database, which summarized genes involved in oxidative stress from validated experimental studies (Supplementary Table S2). The AUCell R package was used to determine each cell line’s oxidative stress AUC activity based on the differential expression level of oxidative stress-related genes in each cell type. Cells expressing higher oxidative stress AUC values in HCC samples were mainly in T memory cells, dendritic cells, and malignant hepatocytes, colored in yellow (Figures 3A, B). As mucosal-associated invariant T cell, T memory cell, and gamma delta T cell clusters were remarkably decreased in the non-tumor group, colored in purple. It can be seen that the oxidative stress AUC of the cells was generally higher in the HCC samples than in the non-tumor liver tissues of the control group in the boxplot (Figure 3C). This means that cells in the HCC microenvironment experienced more severe oxidative stress than non-tumor tissues.

Gene set enrichment analyses were performed to explore the biological processes changes of these cell clusters. In the group of HCC, antigen processing via the MHC class II route and extrinsic apoptosis of T memory cells were triggered. Immune cell receptor pathways were blocked in T memory cells Supplementary Figure S5A). In mucosal-associated invariant T cells from the tumor group, peptidase activity, catabolic process, and cellular response to stress were elevated, and innate immune response, adaptive immune response, and defense response were suppressed (Supplementary Figure S5B). The biological function analysis results revealed that the microtubule-based process, apoptotic process, and response to heat were remarkably activated, while the immune response regulating cell surface receptor signaling pathway and cell recognition were inhibited in the gamma delta T cells of the HCC group (Supplementary Figure S5C). The metabolic process was enhanced in dendritic cells of the HCC group, but the response to growth factor, regulation of lymphocyte activation, and cell activation were repressed (Supplementary Figure S5D). The response to endothelial reticulum stress and monocarboxylic acid metabolic process were greatly stimulated in the B cells of the tumor group, whereas cytoplasmic translation and biosynthetic process were significantly suppressed (Supplementary Figure S5E). Compared to non-tumor hepatocytes, malignant hepatocytes have markedly active cell proliferation and differentiation pathways, including ribonucleoprotein complex subunit organization, electron transport chain, and aerobic respiration. In addition, malignant hepatocytes exhibited suppressed lymphocyte activation, immunological response, and defensive response (Supplementary Figure S5F).

### 3.3 Comparison of cell-cell communication between adjacent non-tumor tissues and HCC

**FIGURE 4:**
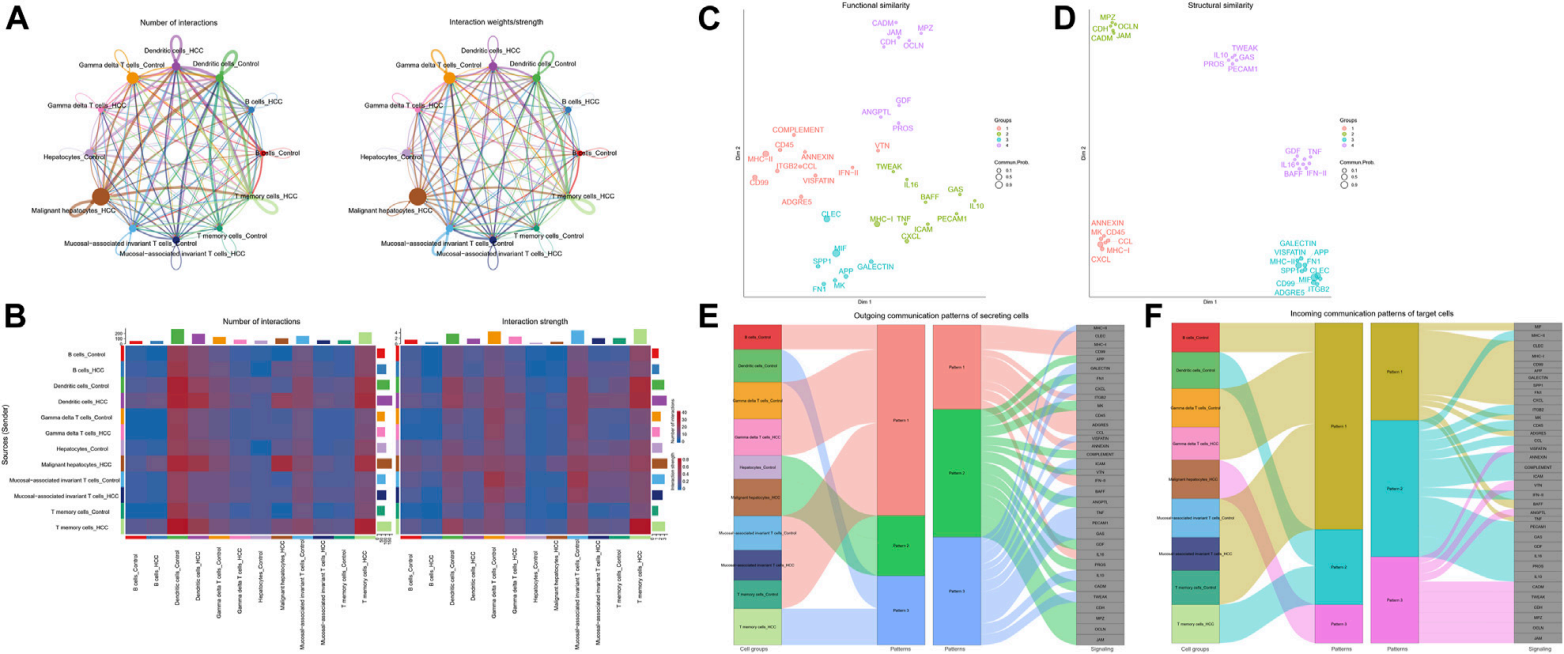
Intracellular communication between HCC and immune cells. **(A)** Interaction of cells in adjacent non-tumor tissue and HCC tissue. The thickness of the line indicates the number of connections or interaction strength, and the arrow indicates the direction of communication. **(B)** Heatmaps displayed the number and strength of the interactions. **(C)** Functional similarity or **(D)** structural similarity classification of shared communication pathways between cells. **(E)** Patterns of functional similarities signaling of secretory cells outcoming or **(F)** incoming to target cells.

Since there were apparent differences in the levels of oxidative stress between HCC cells and adjacent non-tumor tissue cells, we further compared the situations of intercellular communication in the two tissues. According to the preliminary clustered cell-cell communication network analysis results, the interaction strength and net number of interactions between malignant hepatocytes cells and immune cells communication were superior to those between non-tumor hepatocytes cells and immune cells (Figures 4A, B; Supplementary Figure S6). Next, we performed a Laplacian clustering analysis of signaling pathways involved in intercellular communication based on functional and structural similarities (Figures 4C, D). Herein we focus on the functional similarity (two signaling pathways or two ligand-receptor pairs with similar roles) of intercellular communication. Inflammation and immune cell chemotaxis signaling pathways (such as IFN−II, TNF, IL16, IL10, and CXCL) were included in groups 1 and 2. In group 3, MIF, MK, FN1, and Galectin pathways for communication and migration between immune cells and malignant hepatocytes cells. Group 4 mainly involved the intra and exocrine pathways (ANGPTL, GDF) in HCC. Our analysis of signaling pathways in cell populations identified three synergistic patterns of efferent (Figure 4E) and afferent signaling (Figure 4F). The inflammation pathways were the synergistic efferent signaling pathways of T memory cells of adjacent non-tumor tissue and gamma delta T cells, T memory cells, mucosal-associated invariant T cells, and B cells from both tissues. In efferent immune inflammatory response pathways, dendritic cells of adjacent non-tumor tissue and T memory cells of HCC tissue communicate synergistically. Inflammatory pathways were coordinately afferent in T memory cells of adjacent non-tumor tissue, B cells, gamma delta T cells, and mucosal−associated invariant T cells. Dendritic cells of adjacent non-tumor tissue and T memory cells of HCC tissue had synergistic immune chemotactic afferent pathways. Tumor growth and differentiation pathways were coordinately afferent in malignant hepatocytes cells.

**FIGURE 5:**
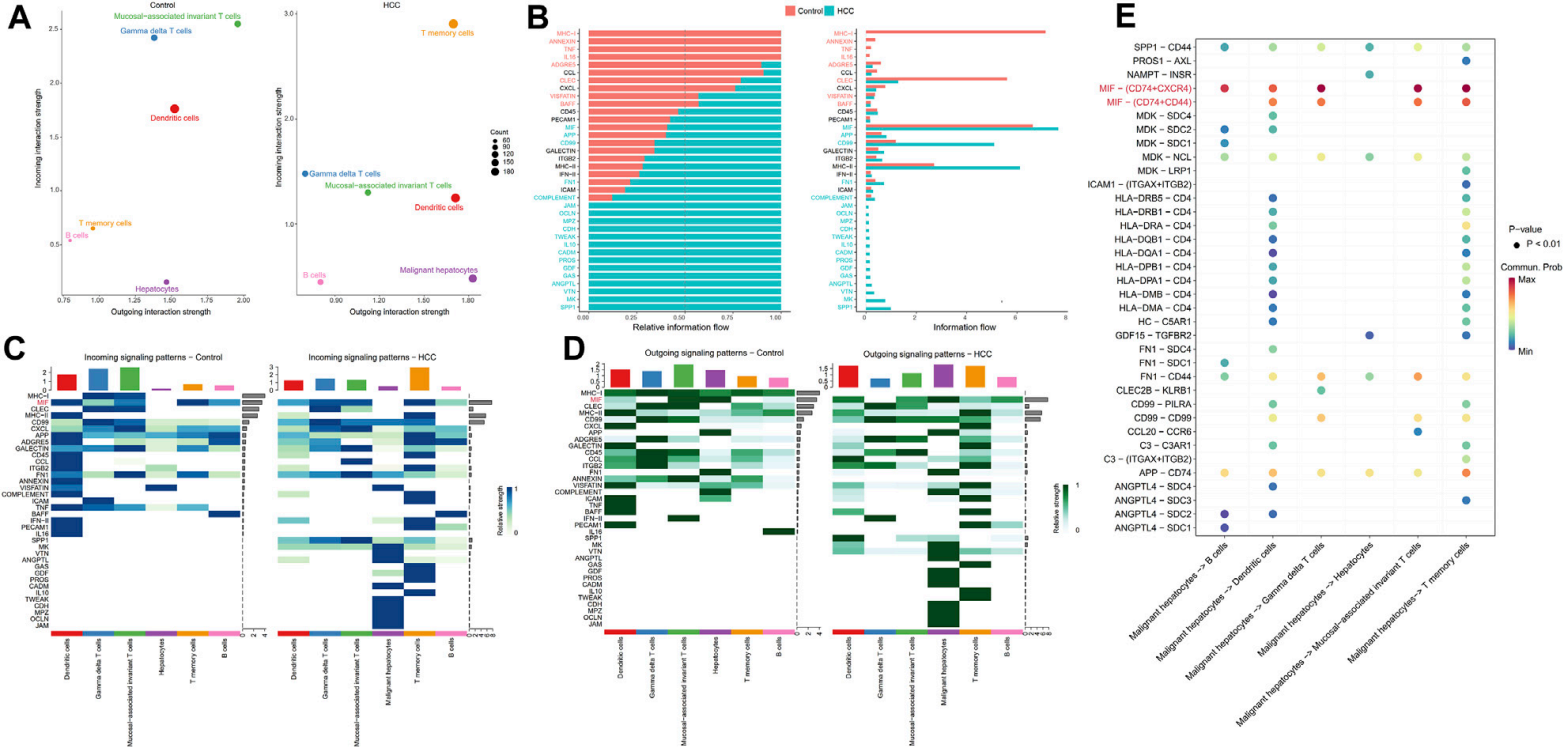
Intercellular communication signaling changes between non-tumor tissue and HCC tissue. **(A)** Cells’ outgoing and incoming interaction strength was analyzed in non-neoplastic and HCC environments. The difference in the size of the dots in the two environments represents the set of cells that have changed sending or receiving signals. **(B)** The information flow changes of signaling pathways between non-tumor and HCC status. **(C)** Incoming and **(D)** outcoming signaling expression differences of cell clusters under the two environments are demonstrated by heatmaps. **(E)** HCC cells have significant ligand-receptor interactions of MIF pathway with immune cells.

**FIGURE 6:**
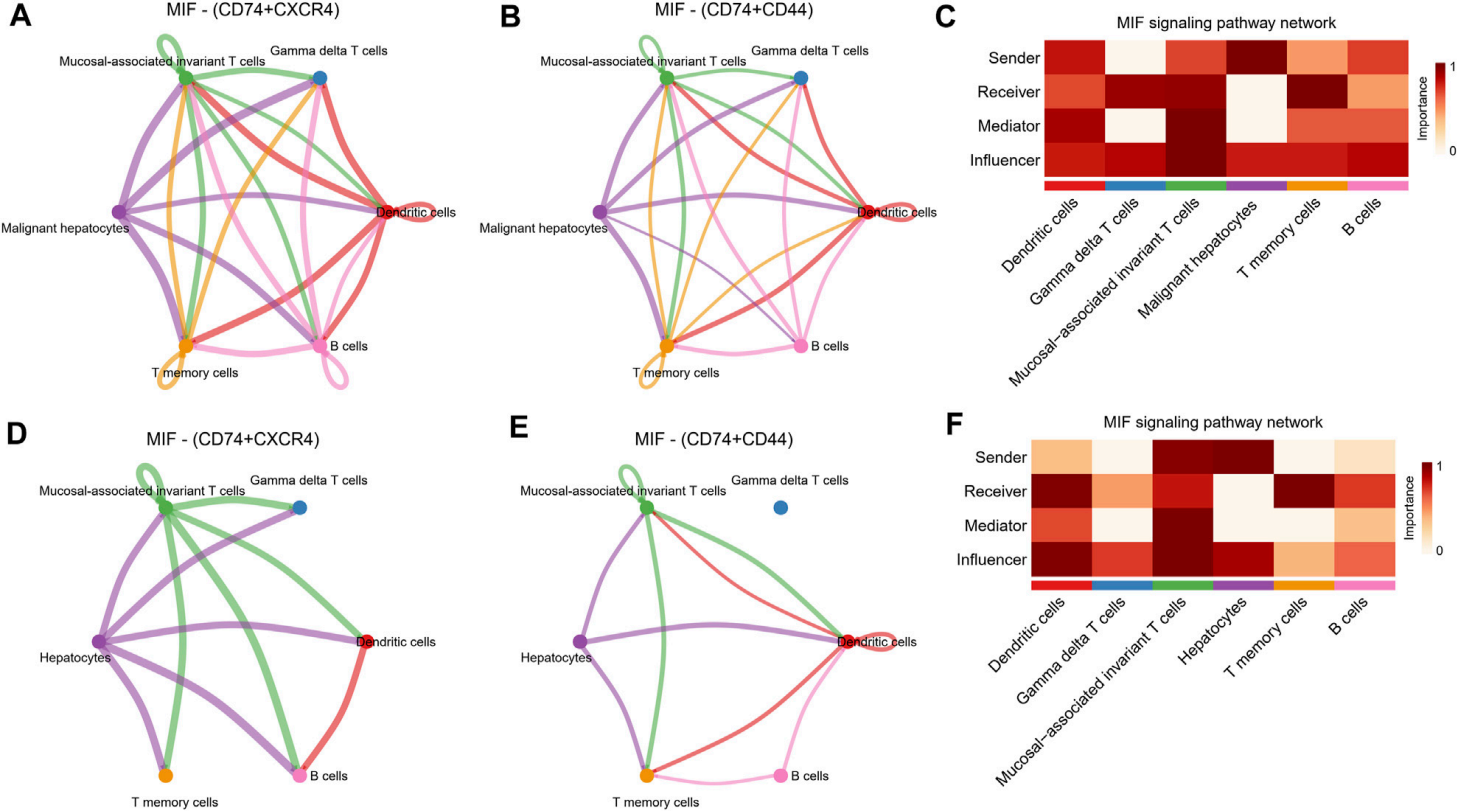
MIF signaling pathway changes between the non-tumor environment and the HCC environment. **(A,B)** Network diagrams of malignant hepatocyte cells communicating with immune cells through ligand receptors of MIF signaling pathway. **(C)** Functional roles in cell-cell communication played by cells in HCC status. The darker the color, the more deterministic the role is. **(D,E)** Network diagrams of non-tumor hepatocyte cells communicating with immune cells through ligand receptors of MIF signaling pathway. **(F)** Functional roles in cell-cell communication played by cells in non-tumor status. The darker the color, the more deterministic the role is.

Further, by comparing outgoing and incoming interaction strength in non-tumor and HCC tissues, we identified significant changes in the signals sent or received by T memory cells, B cells, and malignant hepatocytes (Figure 5A). The memory T cells and B cells in the control group mainly served as signal receivers in cell communication, while the memory T cells and B cells in HCC mainly served as signal transmitters. In HCC, malignant hepatocytes send a higher intensity of intercellular communication signals than non-neoplastic hepatocytes in the control group. The dot plot visually represents the dominant senders and receivers of intercellular communication. The *X* and *Y*-axes correspond to the total outgoing and incoming communication probabilities, respectively, for each cell group. The size of the dots indicates the number of inferred links associated with each cell block, considering both outgoing and incoming interactions. Larger dots indicate a higher number of links. The colors of the dots differentiate between different cell groups, allowing for identification and comparison. Interestingly, we discovered that only the MIF pathway was significantly enriched in non-neoplastic and neoplastic contexts (Figure 5B). Each cell cluster exhibited significant MIF pathway communication in incoming (Figure 5C) and outgoing (Figure 5D) modes under non-tumor and HCC conditions. In light of the above findings, we further investigated the communication between HCC cells and hepatocytes or immune cells through receptor-ligand interaction studies (Figure 5E). The results indicated that malignant hepatocytes and immune cells, including B cells, dendritic cells, gamma delta T cells, mucosal−associated invariant T cells, and T memory cells interact via CD74-CXCR4. Second, HCC cells interact with dendritic cells, gamma delta T cells, mucosal−associated invariant T cells, and T memory cells through CD74^−^CD44. However, no relevant communication between HCC cells and adjacent non-tumor hepatocytes has been found in MIF signaling. Strikingly, MIF communication was significantly more potent in the HCC environment than in the non-tumor environment (Figures 6A, B). The width of the edges in the graph is determined by the number of interactions, which reflects the number of ligand-receptor pairs involved in the communication between two interacting cell clusters. A wider edge indicates a higher number of interactions between the cells. In the HCC state of the MIF signaling pathway (Figure 6C), malignant hepatocytes serve as signal senders. Gamma delta T cells, mucosal−associated invariant T cells, and T memory cells were recipients. Dendritic cells and mucosal−associated invariant T cells act as mediators. B cells and mucosal−associated invariant T cells were the influencers. In contrast, non-neoplastic hepatocytes in the non-tumor state communicate significantly much weaker with immune cells (Figures 6D–F).

### 3.4 Identification of oxidative stress response-related hub genes in HCC

**FIGURE 7:**
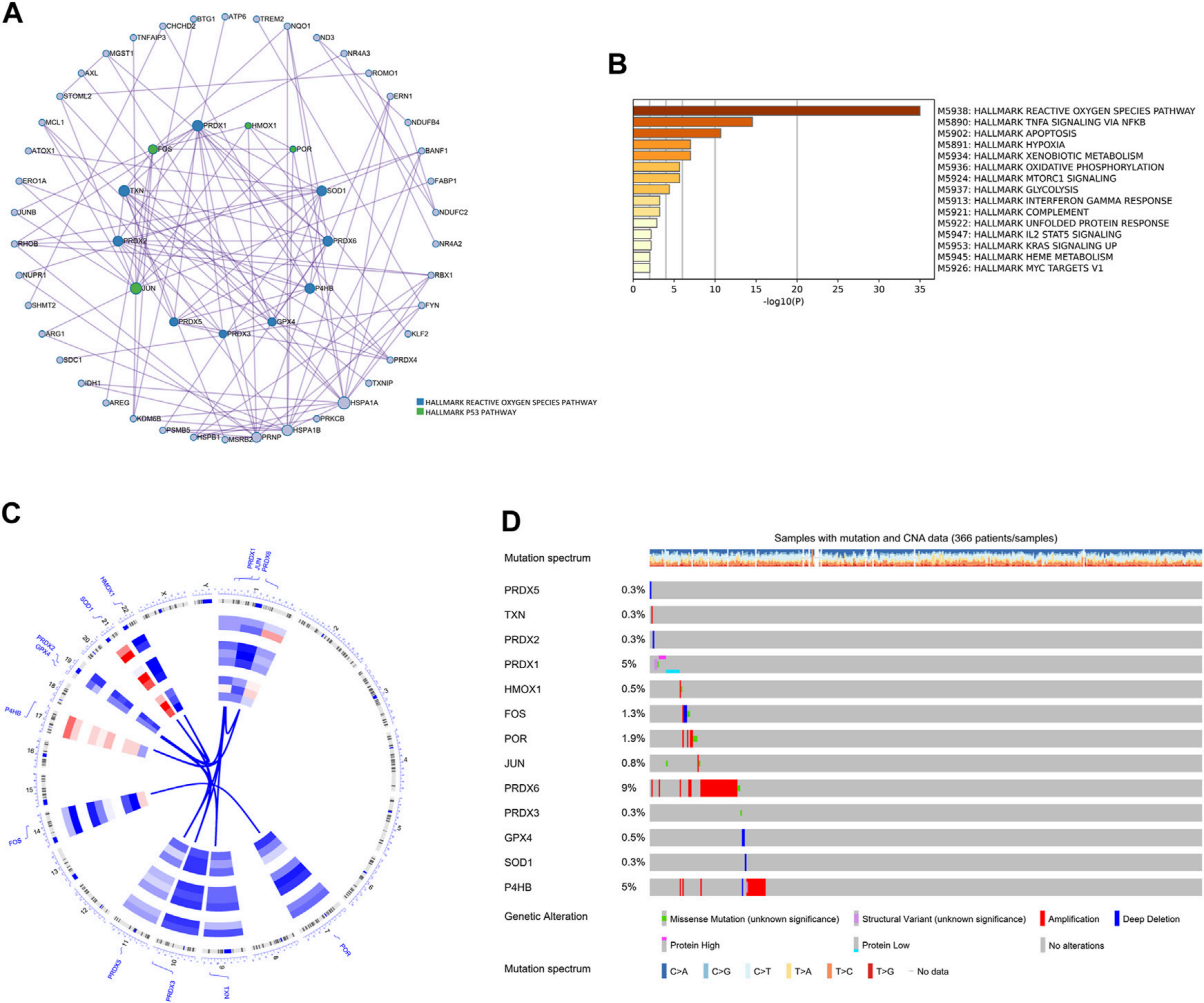
The hallmark pathways and genetic alterations analysis of the oxidative stress-related DEGs in HCC. **(A)** PPI network for hub genes related to oxidative stress among DEGs. Hub genes were colored green and blue. **(B)** The hallmark pathways enrichment analysis of the oxidative stress-related DEGs. **(C)** Circular visualization of chromosomal positions of 13 hub genes. **(D)** The genetic alteration profiles of the 13 hub genes.

Next, differentially expressed oxidative stress response-related genes (DEGs) of each cell type between non-tumor samples and HCC samples were marked in the volcano and bar plot (Supplementary Figures S7A, B; Supplementary Table S3). These DEGs were further subjected to MCODE analysis to screen hub genes of oxidative stress response in the pathogenesis of HCC. The MCODE analysis determined that HMOX1, POR, SOD1, PRDX6, P4HB, GPX4, PRDX3, PRDX5, JUN, PRDX2, TXN, FOS, and PRDX1 were the oxidative stress-related hub genes among DEGs (Figure 7A). Metascape analysis further revealed that the genes HMOX1, POR, JUN, and FOS play a role in regulating the p53 pathway as hub genes. Other hub genes regulate oxidative stress response through the ROS pathway. All the DEGs were associated with the ROS pathway, TNFA signaling via NFKB, apoptosis, hypoxia, and mTORC1 signaling pathway (Figure 7B). This analysis provided evidence of the involvement of these genes in critical biological processes of HCC. Using cBioPortal (Cerami et al., 2012; Gao et al., 2013) tools in the TCGA cohort, we further investigated the mutational landscape of these 13 hub genes in HCC. Chromosome mapping revealed that chromosomes 1 and 19 contain the most significant number of dysregulated oxidative stress-related hub genes in HCC. In contrast, no X and Y chromosomes contain dysregulated oxidative stress-related hub genes (Figure 7C). We analyzed the somatic cell mutation spectrum of 366 patients with HCC to determine the mutation rate of these hub genes. Among the hub genes in HCC patients, PRDX6, P4HB, and POR showed the most significant amplified genetic alteration rates of 9%, 5%, and 1.9%, respectively. PRDX1 displayed a high structural variation rate of 5%. It was observed that FOS had a deep deletion rate of 1.3%. The remaining hub genes exhibited minimal mutation rates below 0.5% in HCC patients (Figure 7D). These findings shed light on the mutational landscape of the hub genes associated with oxidative stress response in HCC, highlighting specific genes with significant genetic alterations.

### 3.5 Screening and verification of diagnostic markers

**FIGURE 8:**
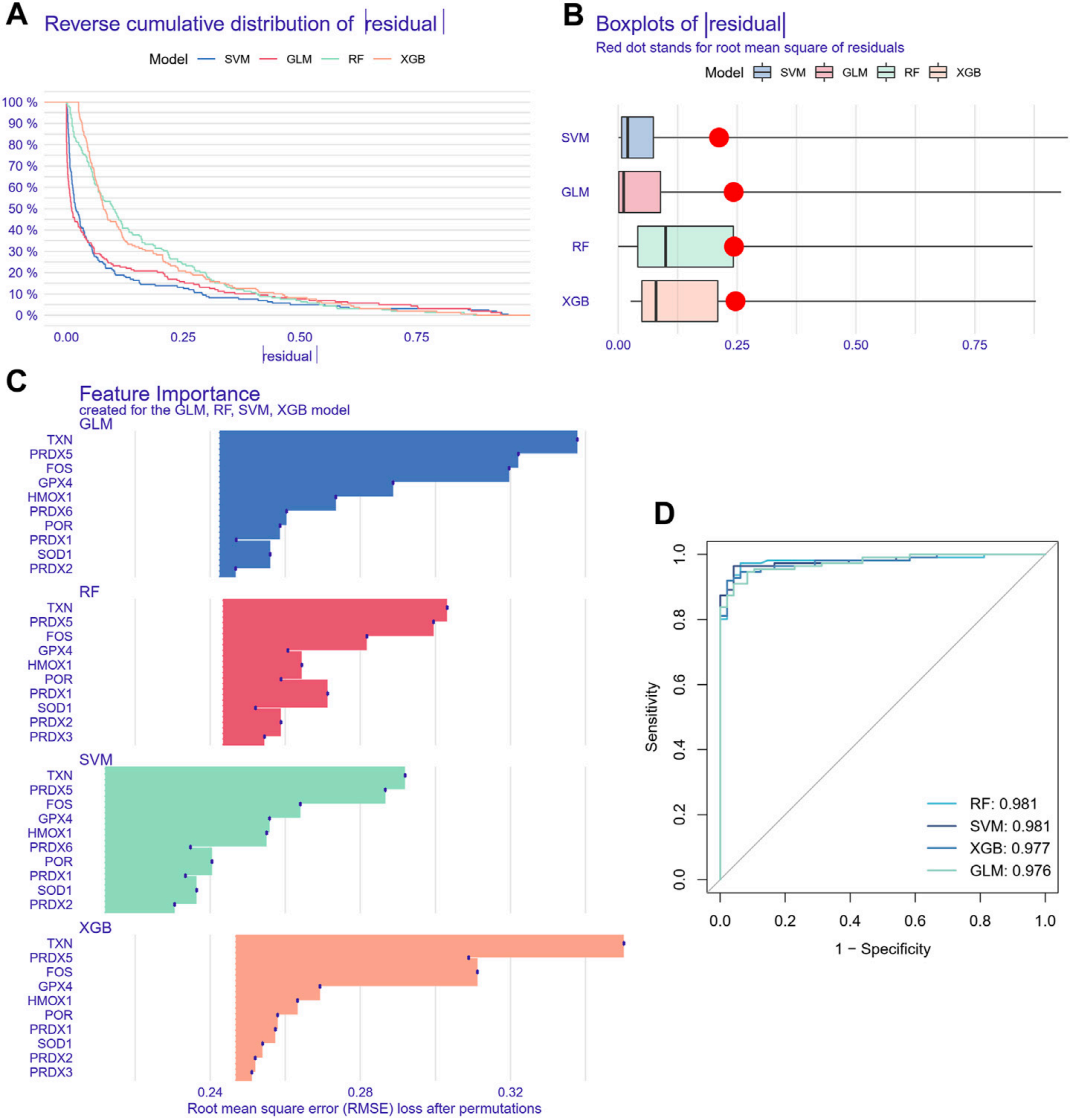
Machine learning model construction and evaluation. **(A)** Each machine learning model’s cumulative residual distribution. **(B)** Boxplots were used to display the residuals of machine learning models. The red dot shows the residuals’ root mean square. **(C)** Genes that are significant in machine learning models. **(D)** ROC analysis for four machine learning models in the testing cohort.

**FIGURE 9:**
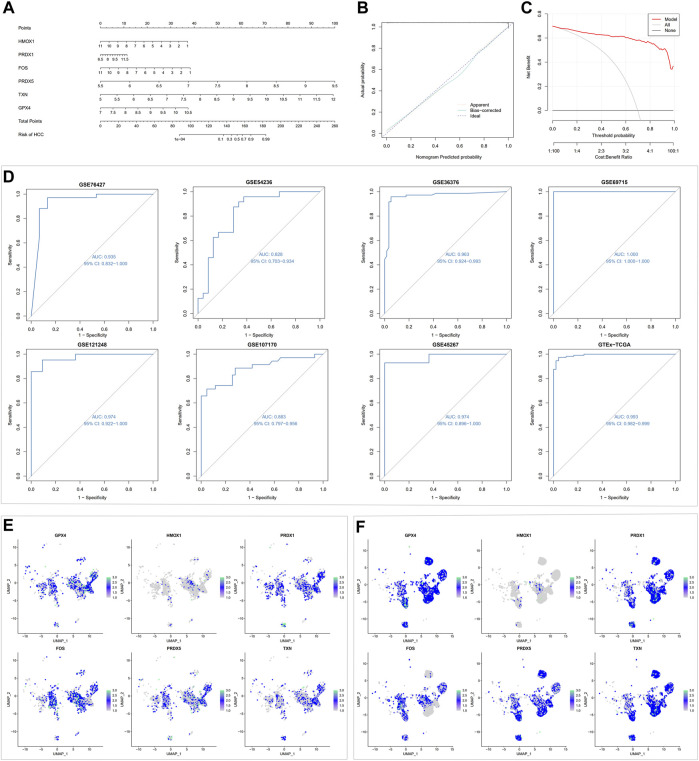
Machine learning-based nomogram model validation. **(A)** Building a nomogram to forecast the risk of HCC. **(B)** Calibration curve analysis for nomogram. **(C)** Decision curve analysis for nomogram. **(D)** ROC analysis for the nomogram model in testing and training cohorts. **(E)** Expression of six gene signatures in non-neoplastic hepatocytes. **(F)** Expression of six gene signatures in malignant hepatocytes.

Since the oxidative stress response level was significantly different between non-tumor tissues and HCC tissue cells, based on the bulk transcript data, we further analyzed the diagnostic value of oxidative stress response-related hub genes by four machine learning models (RF, SVM, XGB, GLM). The RF and SVM models performed the lowest residual values (Figures 8A, B). Meanwhile, the top 10 ranked gene variables in the four machine learning models were filtered out by root mean square error (Figure 8C). By visual ROC analysis, the RF and SVM models also had the highest AUC (0.981) in 5-fold cross-validation (Figure 8D). Then, in conjunction with these outcomes, six oxidative stress response-related gene markers (GPX4, HMOX1, PRDX1, FOS, PRDX5, and TXN) with the highest diagnostic value were chosen from the RF and SVM model (Supplementary Table S4). Further, we constructed a diagnostic nomogram based on these six gene markers to test their clinical decision-making efficiency in predicting the early occurrence of HCC (Figure 9A). The calibration curve analysis showed that the accuracy of the nomogram model in predicting the occurrence risk of HCC was very close to the actual sample probability (Figure 9B). The decision curve analysis reveals that this nomogram is highly accurate with a considerable net benefit (Figure 9C). Additionally, the effectiveness of our nomogram model and gene markers has been verified in seven external datasets by ROC analysis (GSE76427, AUC: 0.935; GSE54236, AUC: 0.828; GSE36376, AUC: 0.963; GSE69715, AUC: 1.000; GSE121248, AUC: 0.974; GSE107170, AUC: 0.883; GSE45267, AUC: 0.974; Training cohort, AUC: 0.993) (Figure 9D, Supplementary Figure S8). The differences in single-cell levels of these six genes between non-neoplastic and malignant hepatocytes were consistent with the transcriptome analysis (Figures 9E, F). Additionally, we investigated the expression of these genes in immunohistochemical pathological sections from The Human Protein Atlas (www.proteinatlas.org/) and found that they were highly expressed, further supporting their potential as biomarkers (Supplementary Figure S9). These results suggested that these six gene diagnostic markers can effectively identify early-stage HCC individuals.

### 3.6 Oxidative stress response-related diagnostic marker PRDX1 was associated with prognosis in HCC

We further performed survival analysis for the six oxidative stress-related diagnostic gene markers from above steps to observe their associations with HCC prognostic progression. Among them, the expression levels of HMOX1, PRDX1, and TXN were significantly related to overall survival in univariate Cox regression analysis of the TCGA-LIHC cohort (*p* = 0.004, *p* < 0.001, *p* = 0.029, respectively). Meanwhile, in the HCCDB18 cohort, PRDX1 and PRDX5 were markedly linked with HCC individuals’ overall survival rate by univariate cox regression analysis (*p* = 0.018, *p* = 0.018, respectively). Based on multivariate cox regression in two cohorts (Supplementary Table S5), PRDX1 was identified to be the independent prognostic predictor for HCC (TCGA-LIHC: *p* < 0.001, Hazard ratio = 1.715, 95%Confidence interval = 1.326–2.217; HCCDB18: *p* = 0.018, Hazard ratio = 1.787, 95%Confidence interval = 1.104–2.892). Thus, PRDX1 was selected for further research.

### 3.7 Validation of the prognostic value of PRDX1 in HCC

**FIGURE 10:**
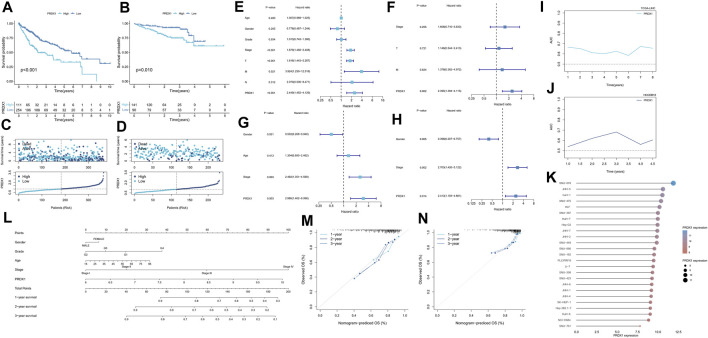
Survival analysis of PRDX1 in the two cohorts. TCGA-LIHC cohort **(A,C,E,F,L,I,M)**, HCCDB18 cohort **(B,D,G,H,J,N)**. **(A,B)** Kaplan–Meier survival analysis result. **(C,D)** Survival status plots of HCC patients in the two cohorts. **(E)** Univariate independent Cox analysis for PRDX1 in TCGA-LIHC cohort. **(F)** Multivariate independent Cox analysis for PRDX1 in TCGA-LIHC cohort. **(G)** Univariate independent Cox analysis for PRDX1 in the HCCDB18 cohort. **(H)** Multivariate independent Cox analysis for PRDX1 in the HCCDB18 cohort. **(I,J)** Time-dependent ROC analysis for PRDX1 in HCC patients. **(K)** Expression profile of PRDX1 in various HCC cell lines. **(L)** The predictive nomogram. **(M)** Calibration curves of nomogram model of the TCGA-LIHC cohort. **(N)** Calibration curves of nomogram model of the HCCDB18 cohort.

Depending on median cut-off values, HCC patients in TCGA-LIHC and HCCDB18 were divided into high and low PRDX1 expression groups. The overall survival of HCC patients in the high PRDX1 group was considerably worse than that of patients in the low PRDX1 group, as shown by Kaplan-Meier analysis (Figures 10A, B). We also found that HCC patients with high expression of PRDX1 have much worse progress-free intervals and disease-specific survival status (Supplementary Figure S10). This tendency of HCC patients was also visible in the survival plot (Figures 10C, D). In univariate cox analysis with clinicopathological characteristics, the PRDX1 was significantly related to the overall survival of HCC patients (TCGA-LIHC: Hazard ratio = 2.449, 95%Confidence interval = 1.452–4.129, *p* < 0.001; HCCDB18: Hazard ratio = 2.988, 95%Confidence interval = 1.462–6.098, *p* = 0.003) (Figures 10E, G). Multivariate cox regression determined that the PRDX1 was an independent predictive factor for patients’ prognoses among clinicopathological characteristics (TCGA-LIHC: Hazard ratio = 2.395, 95%Confidence interval = 1.394–4.115, *p* = 0.002; HCCDB18: Hazard ratio = 2.413, 95%Confidence interval = 1.193–4.881, *p* = 0.014) (Figures 10F, H). At the same time, the prognostic prediction power (AUC) for the PRDX1 in 1 year, 2 years, and 3 years in the ROC analysis of two cohorts was relative satisfaction (Figures 10I, J). Furthermore, a nomogram was created to evaluate the prognosis status of HCC patients by incorporating clinicopathological characteristics, including grade, stage, age, gender, and the expression profile of PRDX1 (Figure 10L). The ideal fitting degree of the calibration curves to the observed values validated the accuracy of nomogram in predicting HCC patients’ outcomes (Figures 10M, N). Moreover, we found the expression level of PRDX1 was consistently high in most HCC cell lines by Cancer Cell Line Encyclopedia (https://sites.broadinstitute.org/ccle) (Figure 10K). As a result, as an independent prognostic factor, PRDX1 has high clinical value in assessing the progression of HCC patients.

### 3.8 Identification pathways and biological functions of PRDX1 in HCC

Gene set enrichment analyses were conducted in HCC, and PRDX1 was found to be mainly involved in digestion, ribonucleoprotein complex biogenesis, ribosome biogenesis, gene silencing by RNA, and smooth muscle cell differentiation (Supplementary Figures S11A, B). Furthermore, we explored the PRDX1-related specific biological process activation status in HCC patients. In the bubble plot, the activated PRDX1-related pathways in HCC patients mainly have ribosome biogenesis, ribonucleoprotein complex biogenesis, ncRNA processing, cellular glucuronidation, uronic acid metabolic process, snRNA processing, and regulation of programmed necrotic cell death (Supplementary Figure S11C). In contrast, PRDX1-related pathways, including vascular-associated smooth muscle cell differentiation, smooth muscle cell differentiation, IL-6 mediated signaling pathway, endosome to plasma membrane protein transport, gene silencing by RNA, and negative regulation of transporter activity, were suppressed in HCC patients.

### 3.9 Correlation between PRDX1 and immune microenvironment in HCC

**FIGURE 11:**
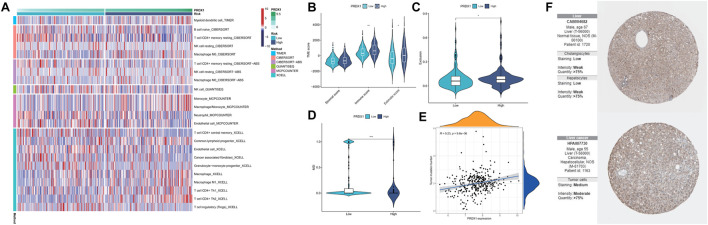
Analysis for immune landscape in two PRDX1 expression groups (*: *p* < 0.05; **: *p* < 0.01; ***: *p* < 0.001). **(A)** The infiltration of immune cells in high- and low-PRDX1 HCC patients. **(B)** Differences in the immune, stromal, and ESTIMATE scores between low- and high-PRDX1 groups. **(C)** T cell exclusion score was different between the two groups. **(D)** Microsatellite instability was different between the two groups. **(E)** The correlation between the TMB and PRDX1 expression level in HCC. **(F)** Immunohistochemical staining intensity for PRDX1 in normal liver tissue and HCC tissue.

**FIGURE 12:**
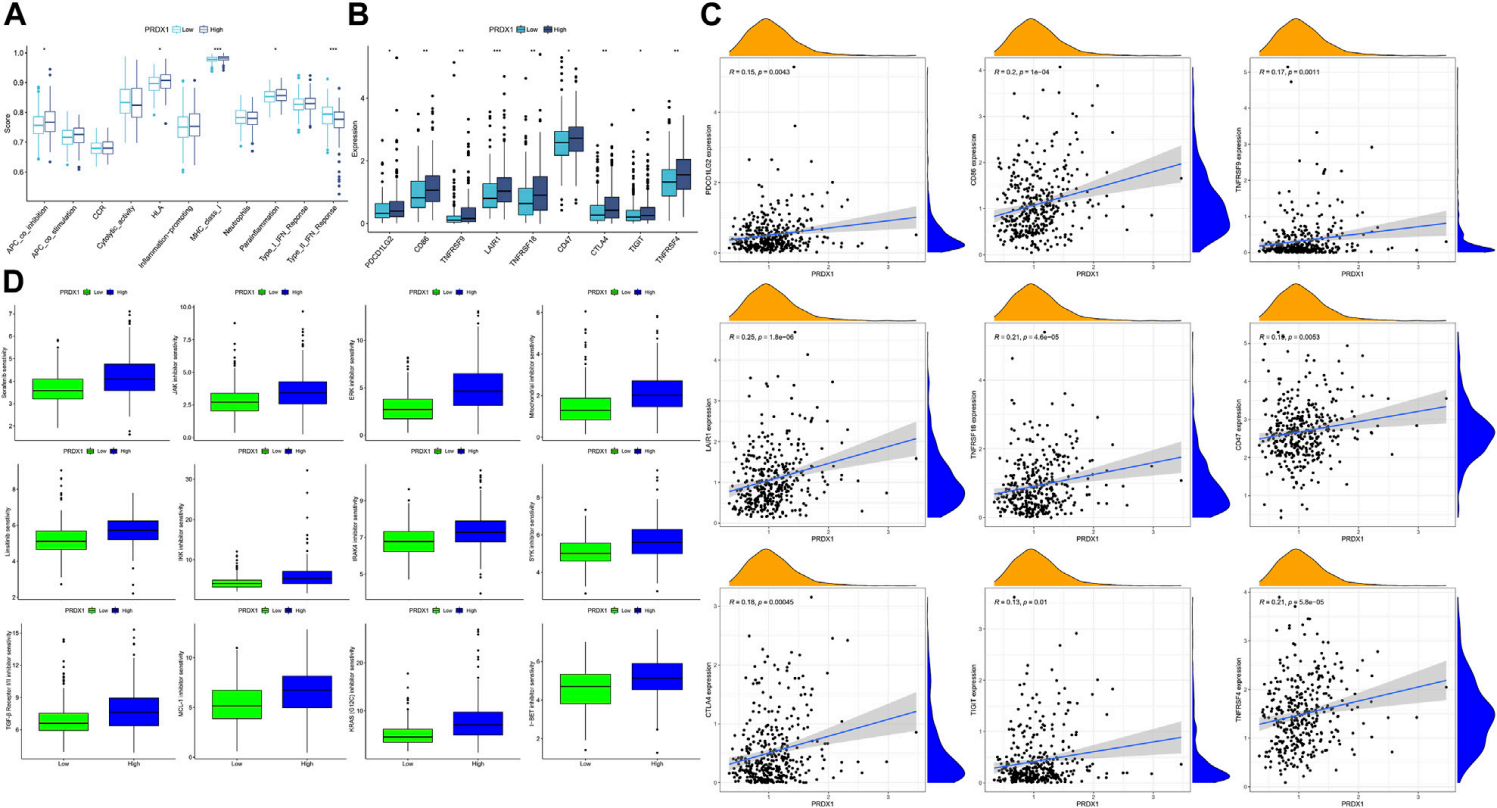
The immune response and immune therapies differences between low-PRDX1 and high-PRDX1 groups (*: *p* < 0.05; **: *p* < 0.01; ***: *p* < 0.001). **(A)** Immune functions analysis in two PRDX1 expression groups. **(B)** Differences of immune checkpoint expression between low- and high-PRDX1 groups. **(C)** The correlation between the expression level of immune checkpoints and PRDX1 in HCC. **(D)** The targeting drug sensitivity in low- and high-PRDX1 groups.

By integrating multiple immune cell infiltration analysis methods (Timer, Cibersort, Cibersort−abs, Quantise, Mcpcounter, and Xcell), the abundances of immune cells, incorporated CD4^+^ T memory resting cell, CD4^+^ T central memory cell, Tregs, neutrophils, myeloid dendritic cell, and NK cells, were markedly enriched in the high-PRDX1 group than the low-PRDX1 group. Non-immune cells, such as endothelial cells, and cancer-associated fibroblast, were also significantly enriched in the high-PRDX1 group (Figure 11A; Supplementary Figure S12). From ESTIMATE algorithm analysis results, the immunological score and estimated score (tumor purity) of HCC patients with PRDX1 high expression were relatively high than those with low expression (Figure 11B). Afterward, the group with high PRDX1 expression held significant T cell exclusion and microsatellite instability (MSI) (Figures 11C, D). Meanwhile, the level of PRDX1 expression linked positively with HCC tumor mutational burden (TMB) (Figure 11E). Moreover, using Human Protein Atlas database (http://www.proteinatlas.org/), we discovered immunohistochemical staining intensity of PRDX1 of the HCC sample was considerably higher than the normal liver sample (Figure 11F). Immune functions, including APC co-inhibition, HLA, MHC class I, and para-inflammation, were active in the HCC patients with high PRDX1 expression. At the same time, type II IFN response was more active in the group with low PRDX1 expression (Figure 12A).

Following, we explored the relationships between immune checkpoints and HCC patients in different PRDX1 expression groups. HCC patients in the high-PRDX1 group had elevated immune checkpoints, incorporating PDCD1LG2, CTLA4, TIGIT, LAIR1, CD86, CD47, TNFRSF9, TNFRSF18 and TNFRSF4 than the patients in the low-PRDX1 group (Figure 12B). Increased expression levels of immune checkpoints were positively correlated with the expression of PRDX1 (Figure 12C). It implied that HCC patients with high PRDX1 expression might benefit more from immune checkpoint inhibitor therapies. To explore suitable drugs for HCC patients with high expression of PRDX1, we used the oncoPredict R package in testing the sensitivity of drugs between two groups with differing PRDX1 expression levels, based on Cancer Therapeutics Response Portal (http://portals.broadinstitute.org/ctrp.v2.1/) and Genomics of Drug Sensitivity in Cancer (https://www.cancerrxgene.org/) databases (Figure 12D). From the results, HCC patients in the high PRDX1 expression group exhibited greater sensitivity to twelve drugs, including Sorafenib (multi-kinase inhibitor), Linsitinib (IGF-1R inhibitor), JAK inhibitor (AZ960), ERK inhibitor (ERK 2440), Mitochondrial inhibitor (Dihydrorotenone), IKK inhibitor (BMS-345541), IRAK4 inhibitor (IRAK4_4710), SYK inhibitor (Entospletinib), TGF-β Receptor I/II inhibitor (LY2109761), MCL-1 inhibitor (AZD5991), KRAS (G12C) inhibitor, and I−BET inhibitor (I-BET-762), than patients with low PRDX1 expression group. These pharmaceuticals could be beneficial in treating HCC patients with high PRDX1 expression.

## 4 Discussion

This present study systematically elucidated the differences in oxidative stress levels between the microenvironment of HCC tissues and the environment of non-tumor tissues as assessed by the AUCell score of the oxidative stress response. The results revealed that oxidative stress response of cells was higher in HCC than in the non-tumor state. Among them, the increased level of oxidative stress response in memory T cells was most notable in the setting of HCC, where antigen processing by oxidative stress and presentation of exogenous peptide antigens via MHC class II was enhanced, and extrinsic apoptosis induced by oxidative stress was also enhanced, but lymphocyte activation function and antigen receptor-mediated signaling pathways were suppressed. This indicated that oxidative stress of immune cells may be a means through which HCC escapes the immune system. In the HCC microenvironment, oxidative stress-induced cell death was attenuated in dendritic cells compared with other immune cells, indicating that it may have a stronger intracellular antioxidant capacity. Current evidence (Hu et al., 2021) shows that oxidative stress is indispensable for dendritic cell functional activation, and ROS-mediated DNA oxidation can enhance immune recognition by dendritic cells, which as a metabolic regulator, can promote STING-dependent dendritic cells’ antitumor immune responses. Clinical treatments with antioxidant intervention may reduce the antitumor activity of dendritic cells. Meanwhile, comparative analysis results between the intercellular communication situation in the HCC microenvironment and that in the non-tumor tissue environment showed that malignant cells communicated more significantly with immune cells through the MIF pathway. Suggesting that more intense levels of oxidative stress in the tumor microenvironment induced MIF secretion to promote HCC cell survival and immunosuppression.

Oxidative stress, a state of oxidative and antioxidant imbalance in the body when it undergoes excessive accumulation of harmful stimuli such as reactive oxygen species, is a critical factor in liver disease progression and hepatocarcinogenesis (Bartsch and Nair, 2006). Several studies have found that the level of oxidative stress in various tumors, such as gastric carcinoma, colon carcinoma, and esophageal carcinoma, is significantly higher than that in non-tumor tissues or healthy tissues, confirming a close relationship between oxidative stress and cancer formation or progression (Dalle-Donne et al., 2006; Agarwal et al., 2012; Bai et al., 2022). It has been summarized that under various pathological conditions, oxidative stress could be induced by ROS generated from electron leakage through mitochondrial electron transport in liver disease, thereby damaging hepatocytes, promoting pathological polyploidization, triggering inflammation, and leading to the activation of oncogenic pathways (Sasaki, 2006; Cichoz-Lach and Michalak, 2014; Higgs et al., 2014; Satapati et al., 2016).

Redox reactions are fundamental processes underpinning critical cellular functions like oxidative phosphorylation for energy production (Nath and Villadsen, 2015). Furthermore, redox changes are instrumental in cell signaling, modulating the activities of enzymes, receptors, and transcription factors through the redox state of specific cysteine residues (Navarro-Yepes et al., 2014). Differentiating between normal redox fluctuations and oxidative stress is nuanced and multifaceted. This distinction hinges on intricate factors, including the type of ROS, their subcellular localization, and the duration of exposure. It is important to note that transient increases in ROS during immune responses or cellular differentiation are often essential for these processes and do not constitute oxidative stress (Forman et al., 2010). Cellular redox balance is maintained through the collaborative efforts of enzymatic and non-enzymatic antioxidants (Halliwell and Gutteridge, 1986). The equilibrium between ROS and antioxidants dictates whether a redox shift remains adaptive or evolves into oxidative stress. Defining oxidative stress lacks a universal threshold due to its context-dependent nature. Researchers (Pouvreau, 2014; Wu et al., 2019; Zaidieh et al., 2019) have employed various indicators such as the glutathione redox ratio, ROS concentrations, or specific oxidative damage products. These thresholds can differ based on cell types, tissues, and disease conditions. Transitioning from a redox change to oxidative stress carries profound biological implications. Oxidative stress can damage lipids, proteins, and nucleic acids, resulting in mutations, cellular dysfunction, and the initiation of inflammatory cascades (Harrington et al., 2023).

The basal oxidative state is a cornerstone of cellular biology, serving as a linchpin in cellular signaling. ROS act as vital signaling molecules when present in controlled quantities, participating in cell proliferation, differentiation, and apoptosis processes (Bae et al., 2011). Key transcription factors like NF-κB and Nrf2, which modulate inflammation and antioxidant defenses, are sensitive to redox changes. Across diverse cell types and organelles, the basal oxidative state exhibits variations. For example, renowned for their redox activity, mitochondria meticulously regulate ROS production and elimination to optimize their function (Rigoulet et al., 2011). Typically, intracellular ROS concentrations fall within the picomolar to low nanomolar range, underscoring the necessity for precise regulation (Gupta et al., 2014). Cells employ a complex network of antioxidant systems to preserve the basal oxidative state. This network encompasses enzymatic antioxidants and non-enzymatic antioxidants. These antioxidants collaborate to scavenge surplus ROS and uphold redox equilibrium. Departures from the basal oxidative state can have far-reaching consequences for cellular processes. Elevated ROS levels can lead to oxidative damage to biomolecules, inducing DNA mutations, protein misfolding, and lipid peroxidation. Conversely, an overly reduced environment can disrupt redox-sensitive signaling pathways, impair immune responses, and contribute to disease pathogenesis (Nakamura et al., 1997). Dysregulation of redox balance underpins a multitude of pathological conditions. Chronic oxidative stress is implicated in cancer. A comprehensive understanding of and interventions targeting the basal oxidative state hold profound clinical implications for disease management and prevention.

To further explore the possible impact of oxidative stress levels on cellular crosstalk, we contrasted the strength of intercellular communication in a non-tumor environment with low *versus* a tumor environment with higher levels of oxidative stress. Interestingly, in an analysis of intercellular communication, this study found that the communication strength between malignant and immune cells in the MIF pathway was significantly greater in the setting of HCC with higher levels of oxidative stress than in the setting of non-tumor hepatocytes. MIF is a pleiotropic cytokine overexpressed in many tumors and has pro-inflammatory and tumor-promoting activities (Bucala, 2012). Studies have shown that MIF can affect the migration of tumor cells and inhibit immune cell infiltration into tumor tissue, affecting tumor cells and tumor stroma through multiple mechanisms (Simpson et al., 2012; Pasupuleti et al., 2014). There were evidences that a variety of immune and non-immune cells, including T cells, macrophages, dendritic cells, and epithelial cells, upon stimulation by noxious factors such as hypoxia and UV irradiation, secretes MIF (Sakamoto et al., 2002; Simons et al., 2011; Heise et al., 2012; Merk et al., 2012). Currently, it is unclear how MIF is modulated in tumor. The complexity of MIF in cancer emphasizes the necessity of gaining a deeper understanding of its biological function. Gupta et al. (2016) found that the regulation of p53 did not induce MIF secretions in renal, breast, and lung cancerous cells, but oxidative stress was a mediator of the stimulator of MIF secretions. The CD74^−^CD44 receptor complex and the chemokine receptor CXCR4 activated MIF signaling, which in different types of cells activates both anti-apoptotic and pro-survival pathways via MAPK, Akt, and SRC pathways (Leng et al., 2003; Shi et al., 2006; Bernhagen et al., 2007; Mitchell and Yaddanapudi, 2014). Takahashi et al. 1(998) found that inhibition of endogenous MIF expression could slow the growth rate of tumor cells, and knockdown of MIF inhibited HCC cells proliferation. Wirtz et al. (2021) experimentally demonstrated that inhibition of the MIF/CD74 axis, which induces HCC cell death and activates ERK, can reduce the number of tumors and the proliferation rate. These are consistent with our findings. Although some progress has been made in the current study on MIF regulation of immune responses and inflammatory responses in cancer, its biological functions related to oxidative stress need to be further explored.

Based on the close relationship between oxidative stress and tumorigenesis and the significant difference in oxidative stress between HCC tissues and HCC tissues, we analyzed the hub genes of oxidative stress-related DEGs in cells to provide more clues for clinical decision-making. The thirteen oxidative stress-related hub genes are HMOX1, POR, SOD1, PRDX6, P4HB, GPX4, PRDX3, PRDX5, JUN, PRDX2, TXN, FOS, and PRDX1. They were significantly enriched in TNF, apoptosis, hypoxia, and mTOR pathways, in addition to their significant expression in ROS pathways. Previous studies have revealed that machine learning with multivariate analysis, which considers the correlations between factors, has a lower error rate than univariate analysis and can vastly boost the precision of forecasting tumor susceptibility (Cruz and Wishart, 2007; Painuli et al., 2022). In this study, we included four machine learning models (RF, GLM, SVM, and XGB) validated to have robust classification ability (Tan et al., 2014; Rigatti, 2017; Zopluoglu, 2019; de Melo et al., 2022). Since the RF *versus* SVM model had the equal highest predictive power (AUC = 0.981) in the test cohort, we selected the utmost important six feature gene variables (GPX4, HMOX1, PRDX1, FOS, PRDX5, and TXN) in both models. Then we constructed a nomogram model for the clinical diagnosis of HCC based on these six genes. In both the calibration curves and the evaluation in three external validation sets (AUC = 0.974, 0.883, 0.974), our nomogram model effectively predicted HCC. Therefore, the nomogram obtained by screening six oxidative stress-related genes through multiple machine learning algorithms was a reliable indicator for evaluating the pathological outcomes of patients, providing new insights into the potential role of oxidative stress in the clinical diagnosis of HCC.

In a further analysis, we found that PRDX1, among these six oxidative stress-related genes in the nomogram, was strongly associated with the prognosis of HCC and could contribute to judging the prognosis of HCC patients. Individuals with HCC who had high PRDX1 expression exhibited shorter overall survival, progression-free interval, and disease-specific survival time. Additionally, we found that PRDX1 was ubiquitously highly expressed in multiple liver cancer cell lines. PRDX1 is a multifunctional protein involved in cell proliferation, differentiation, and apoptosis that belongs to the peroxidase family and whose function in cancer is not well understood (Sun et al., 2022). It has been shown that overexpressed PRDX1 can activate toll-like receptor4, mTOR pathway, and TGF-β1 promotes tumor development and progression (Riddell et al., 2012). The single gene functional GSEA analysis of PRDX1 in HCC patients revealed a strong association between PRDX1 and the digestive system. PRDX1-related regulation of ribosome biogenesis, ncRNA processing, and snRNA processing was hyperactivated in HCC patients. In contrast, the PRDX1-associated IL-6-mediated signaling pathway, RNA gene silencing, and negative regulation of transporter activity were suppressed. Recent evidence (Elhamamsy et al., 2022) has revealed that ribosome biogenesis is a central process that promotes cell survival and stress-adaptive responses, collectively triggering tumor initiation and metastasis through ribosome modification. Cancer cells contain specialized oncogenic ribosomes that promote the translational program of oncogenes, regulate cellular functions and promote metabolic restructuring, increasing the risk of malignancy. Inferring low expression of PRDX1 may have an inhibitory effect on tumor progression.

For now, there are relatively few studies on the involvement of PRDX1 in the immunological microenvironment of HCC, which was analyzed by bioinformatics in our study. In the tumor microenvironment, HCC patients with low PRDX1 expression had more significant infiltration levels of M0 macrophages, M1 macrophages, CD4^+^ Th1 cells, CD4^+^ Th2 cells, myeloid dendritic cells, monocytes, and lymphoid progenitor cells. Whereas the endothelial cells and cancer associated fibroblasts were more abundant in the poor prognosis PRDX1 high expressing HCC patient population. Microsatellite instability, tumor mutational burden, and T cell exclusion are more pronounced in HCCs with high PRDX1 expression. Relevant immune functions APC co-inhibition, MHC class Ⅰ, and para-inflammation were more active in patients with high PRDX1 expression. Type Ⅱ INF response was more active in the low PRDX1 expression patient group. The expression levels of immune checkpoints (as PDCDL1G2, LAIR1, CTLA4, TIGIT) were positively correlated with the expression level of PRDX1. Our analysis of the potential role of PRDX1 in clinical therapeutic intervention revealed that HCC patients with high PRDX1 expression were susceptible to immunotherapy agents such as sorafenib, JAK inhibitor, IRAK4 inhibitor, and TGF-β Receptor I/II inhibitor, SKY inhibitor, and ERK inhibitor have a high degree of sensitivity. These findings indicated that PRDX1 is a prospective biomarker for predicting the prognosis and guiding treatment decisions for HCC.

However, this study relying on single-cell data from public databases have limitations that constraints on sample size and cell numbers and incomplete coverage of cell types and conditions. Therefore, the results may be affected by the inherent bias in case selection. Further large-scale prospective studies and *in vivo* and *in vitro* experiments were required.

## 5 Conclusion

In conclusion, our study unveiled variations in oxidative stress levels between non-tumor liver and HCC tissues. And we identified oxidative stress gene markers associated with hepatocarcinogenesis development, offering novel insights into the oxidative stress response mechanism in HCC.

## Data Availability

The original contributions presented in the study are included in the article/Supplementary Material, further inquiries can be directed to the corresponding authors.
