# Supplementary figures and images for: Single cell and bulk transcriptome analysis identified oxidative stress response-related features of Hepatocellular Carcinoma
Source: Front Cell Dev Biol. 2023 Sep 28;11:1191074. doi: 10.3389/fcell.2023.1191074 (PMC10568628; doi:10.3389/fcell.2023.1191074)

**A** CIBERSORT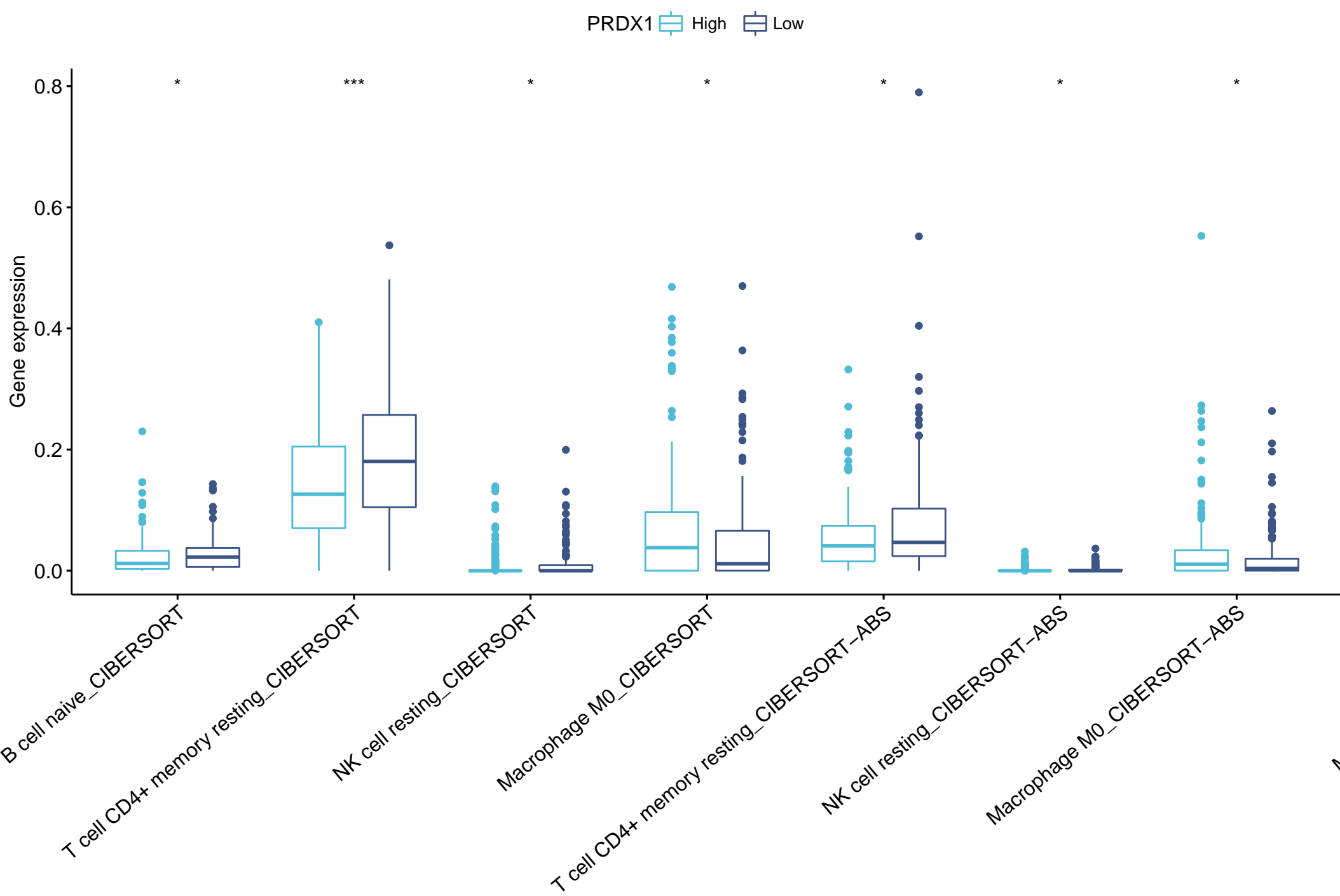**B** MCPCOUNTER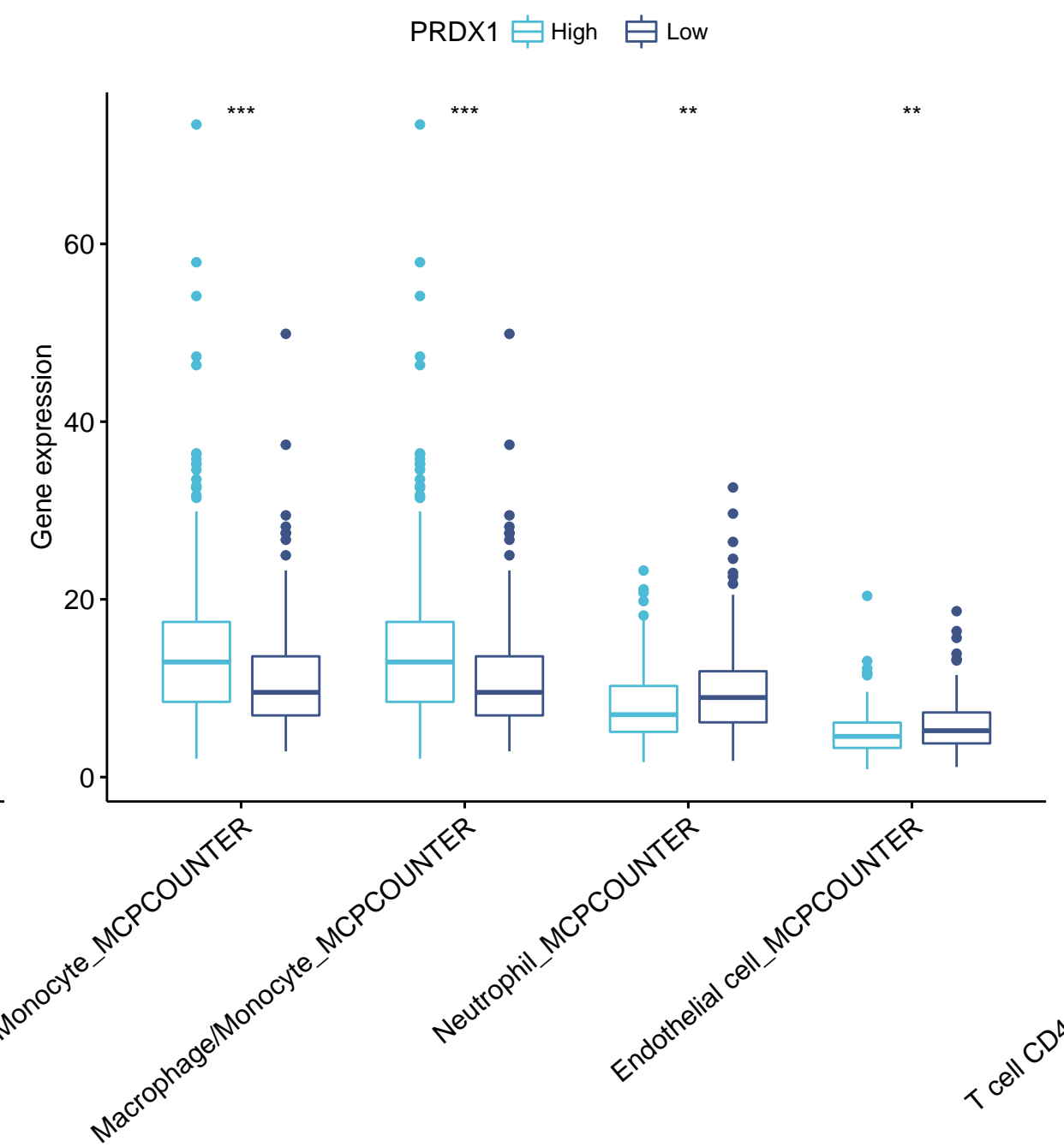**C** XCELL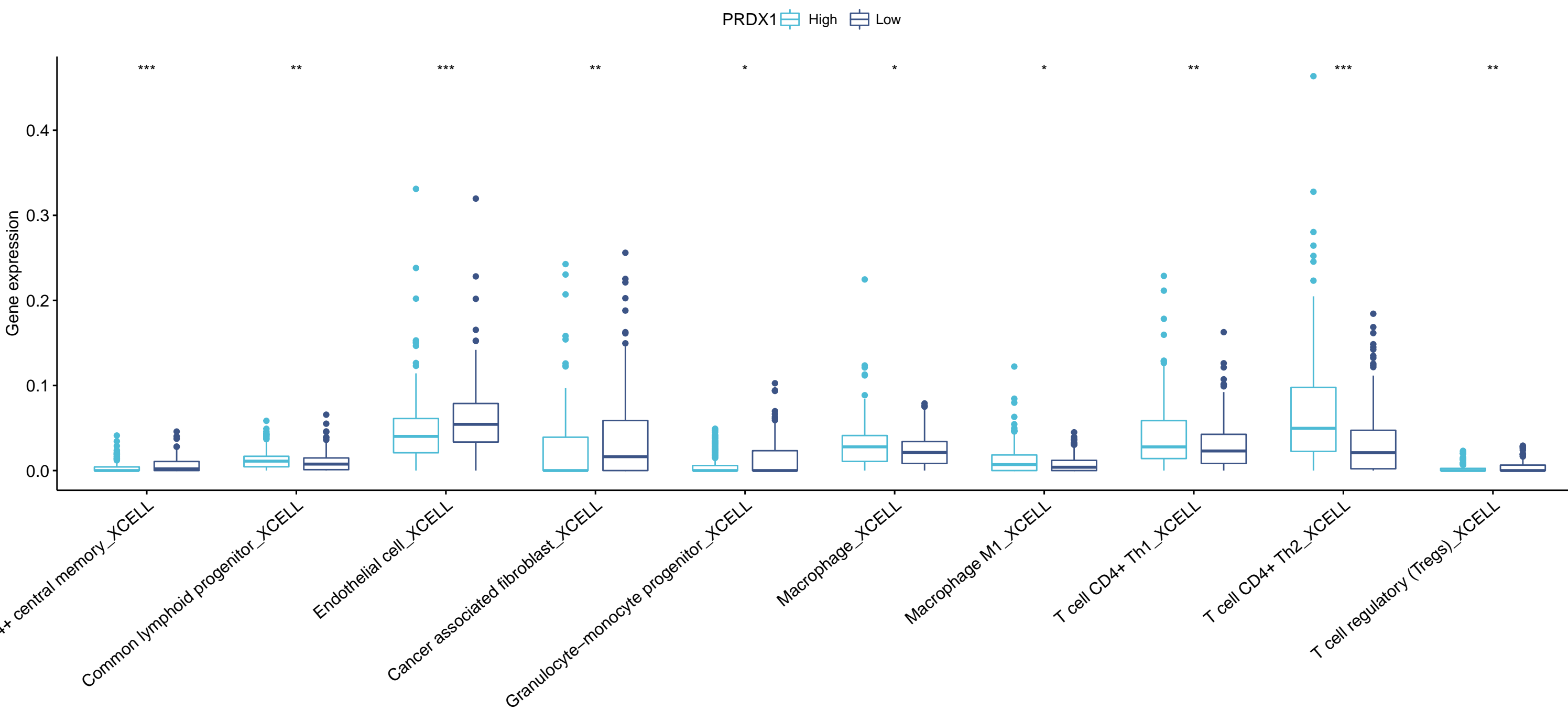**D** TIMER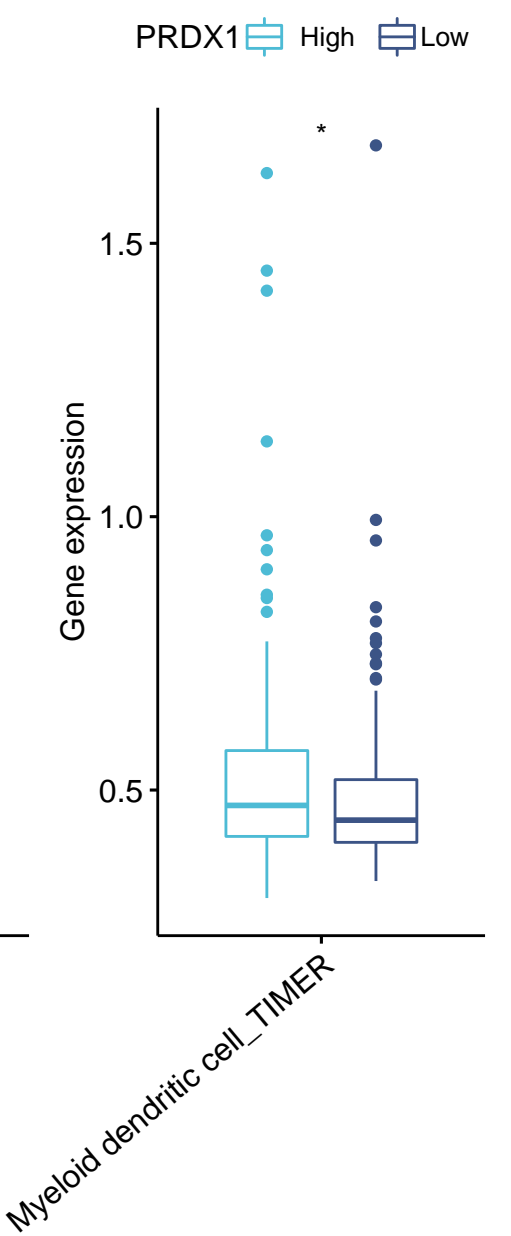**E** QUANTISEQ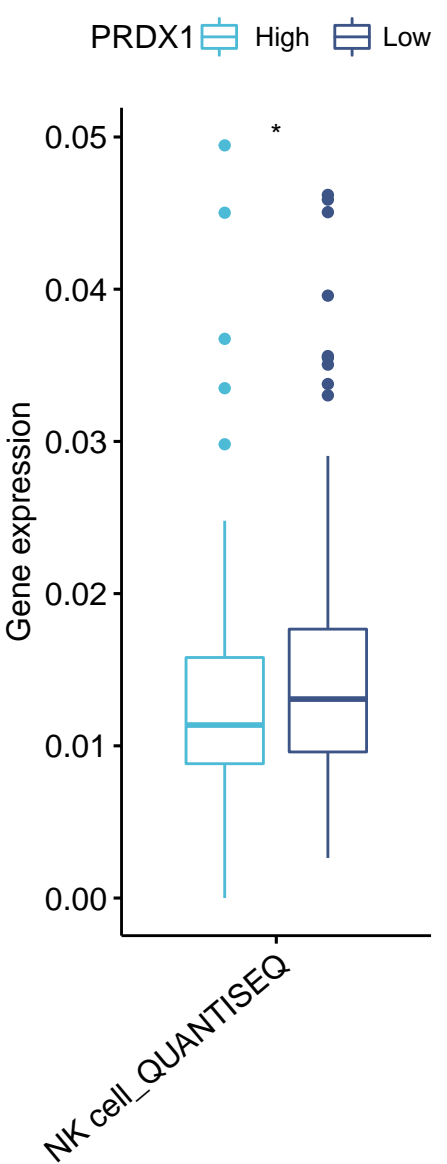

Supplement: Supplementary file 2 [file Image12.PDF]

Survival probability

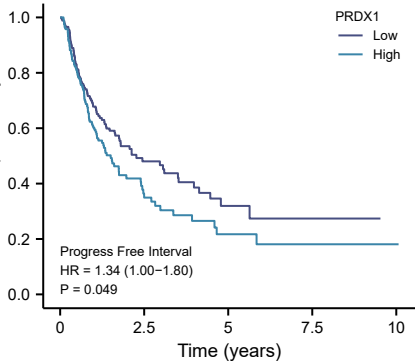

Low

185

39

11

2

0

High

185

24

7

1

1

Survival probability

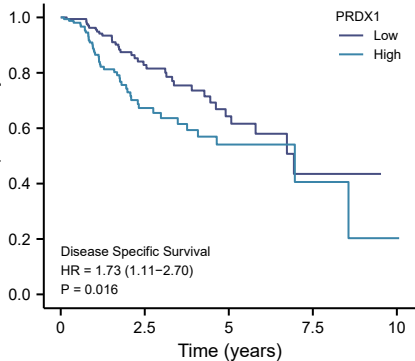

Low

183

66

24

5

0

High

179

41

16

3

1

Supplement: Supplementary file 6 [file Image10.PDF]

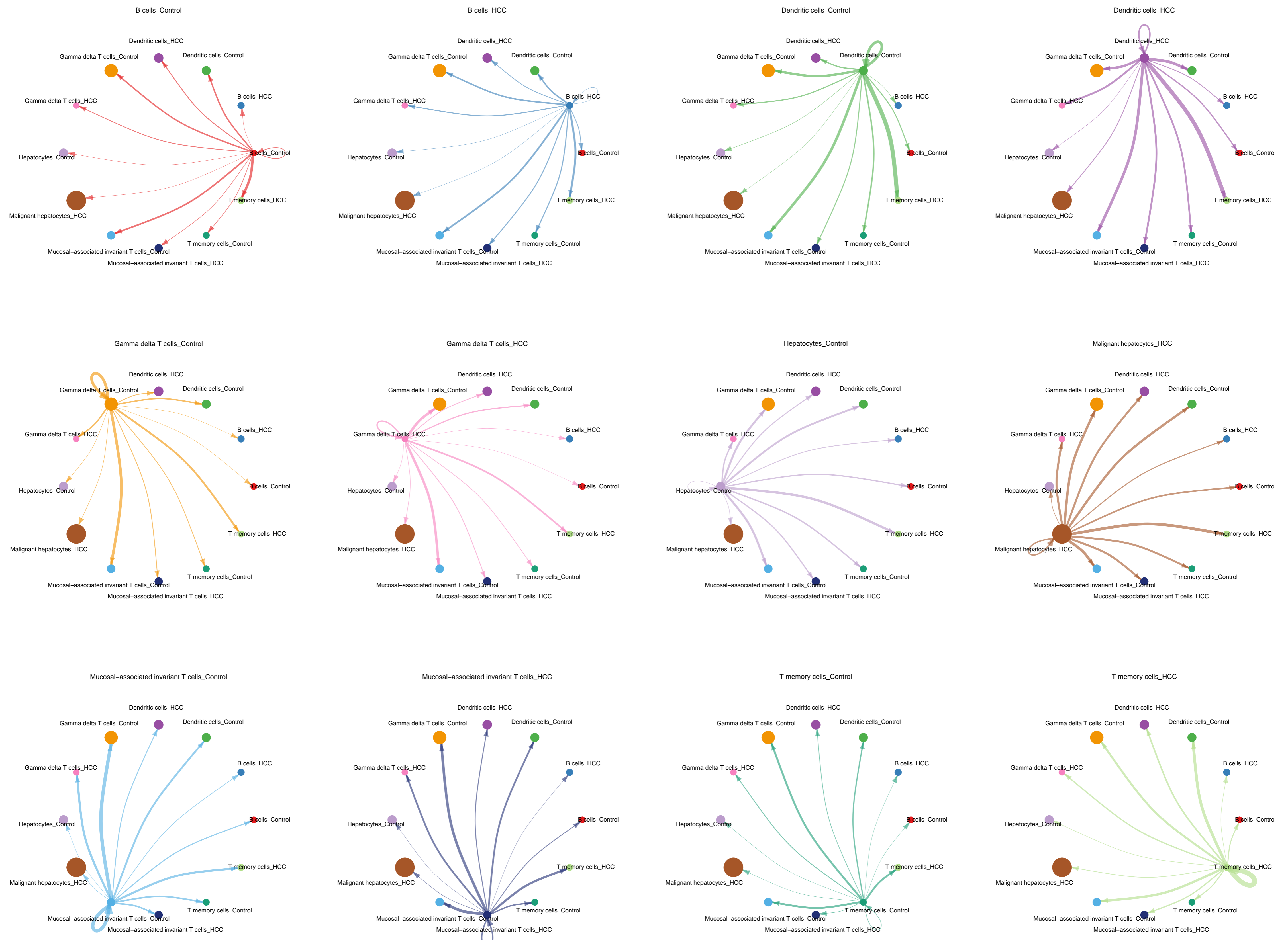

Supplement: Supplementary file 7 [file Image6.PDF]

GSE76427

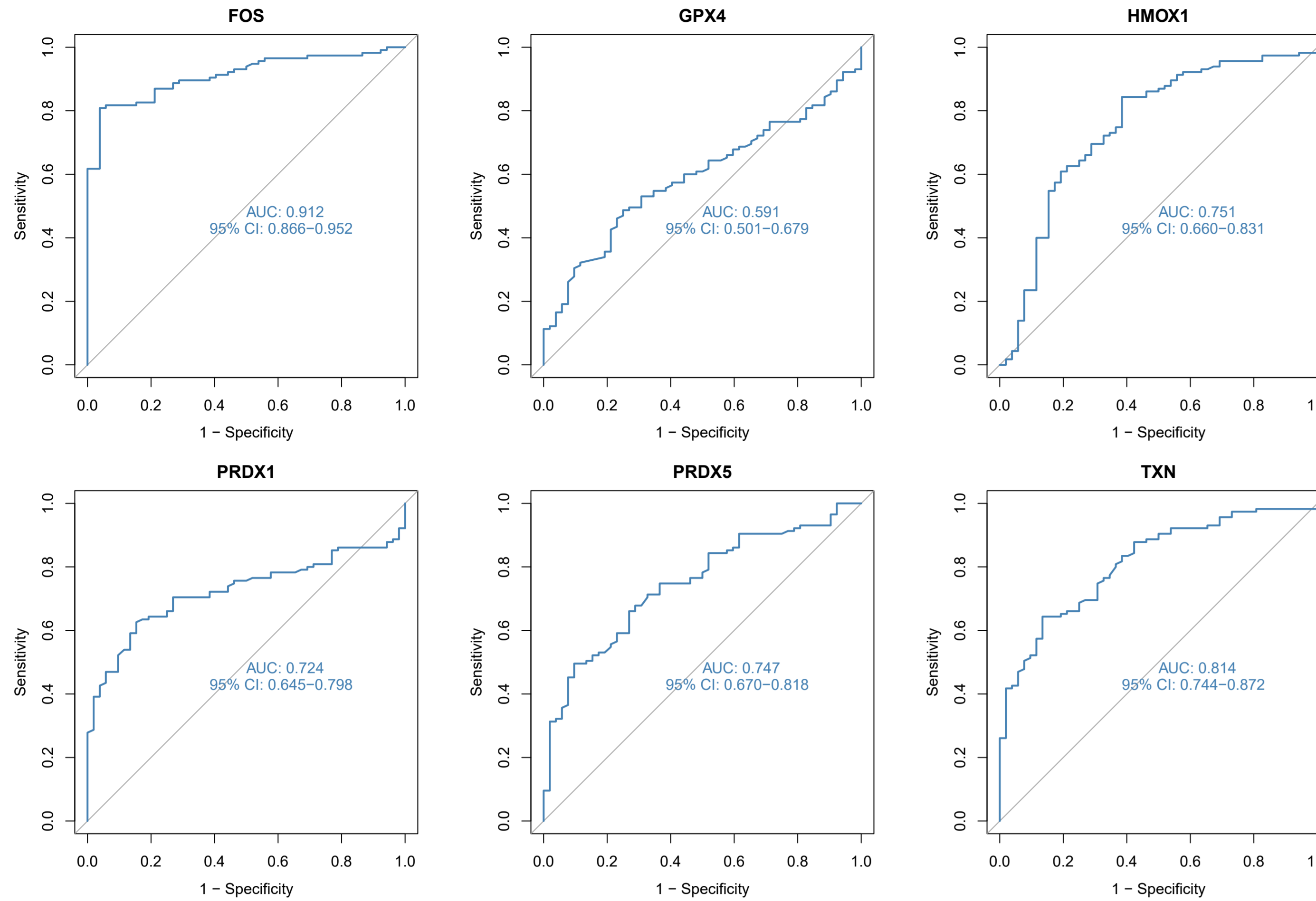

GSE54236

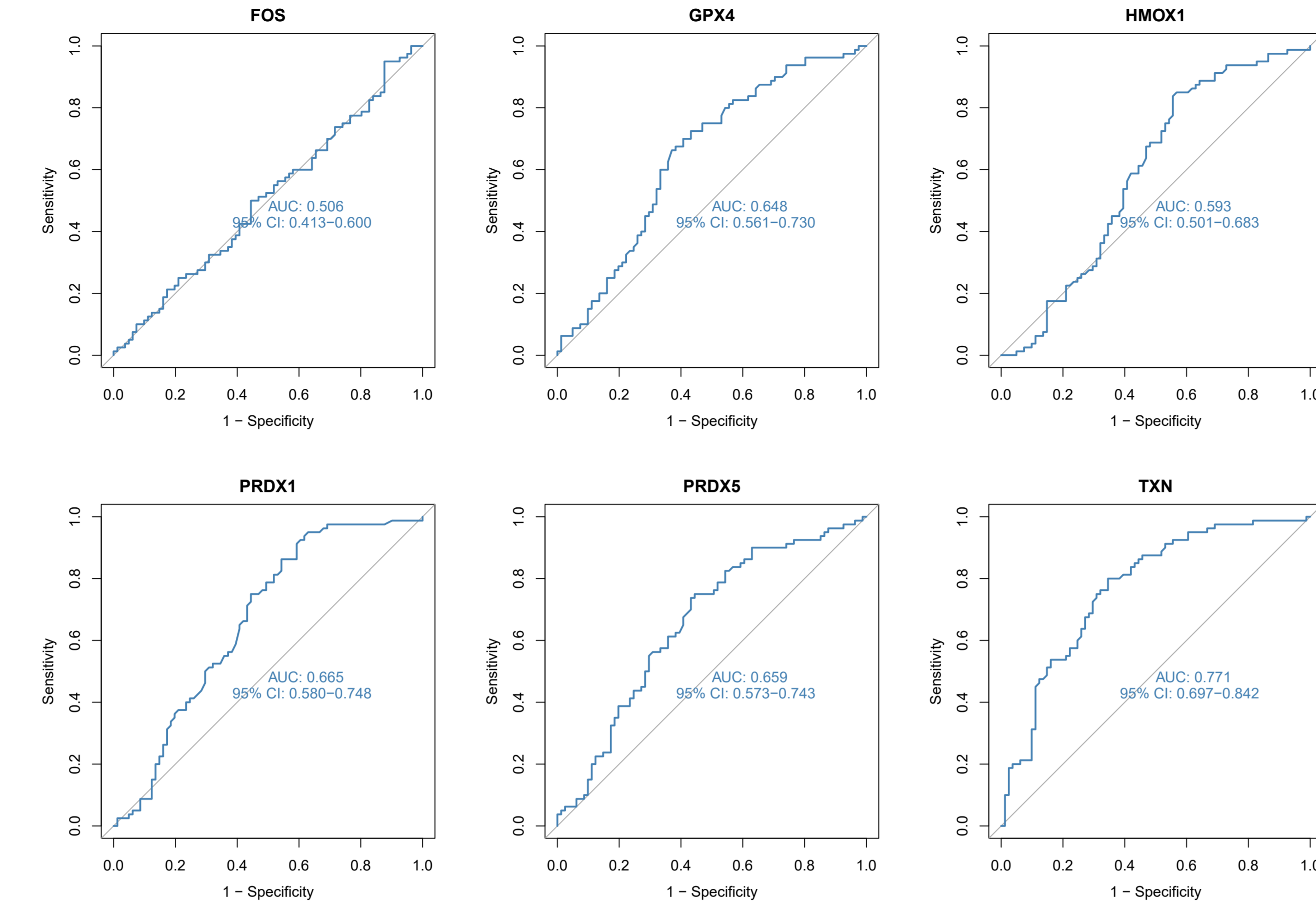

GSE107170

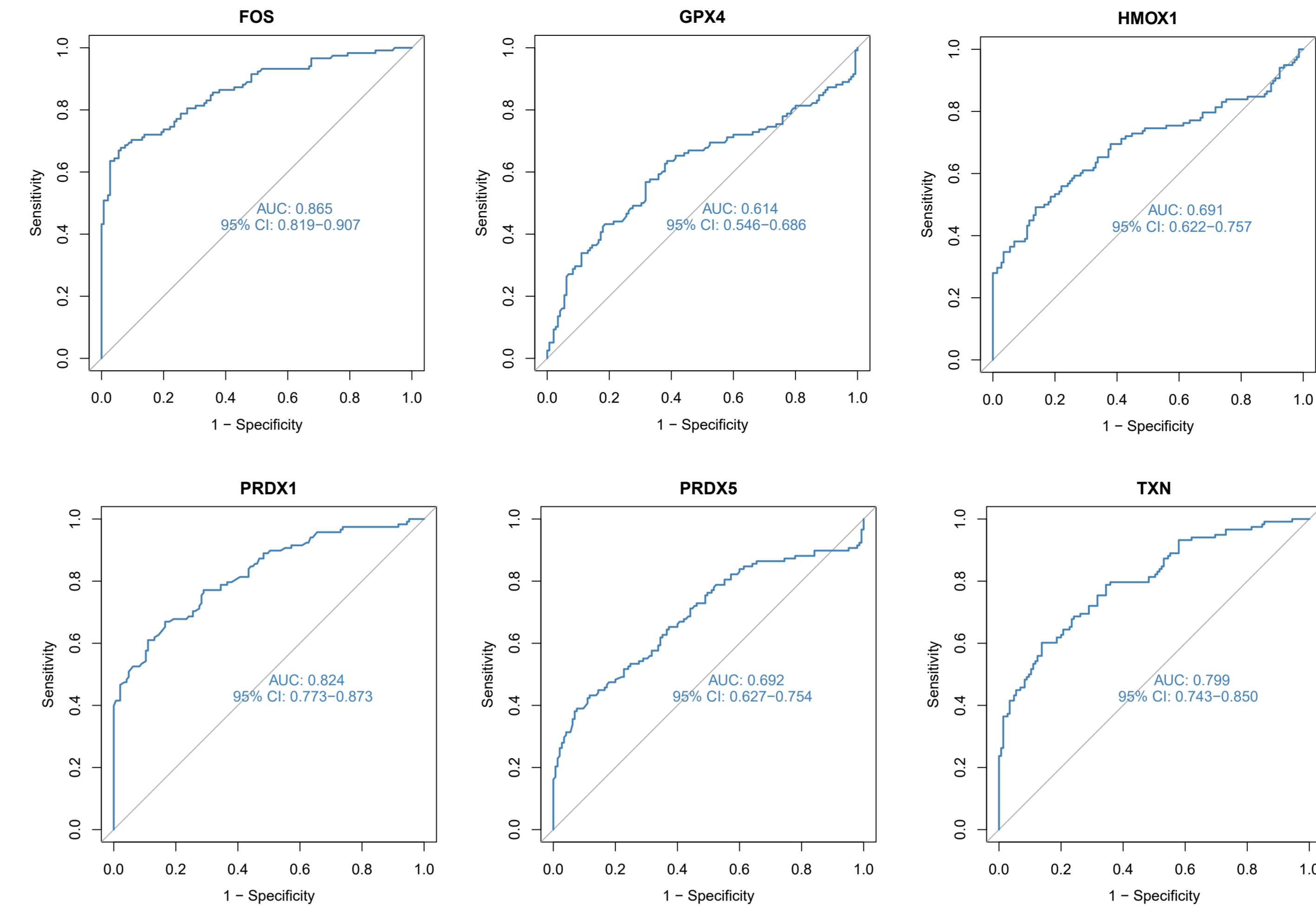

GSE121248

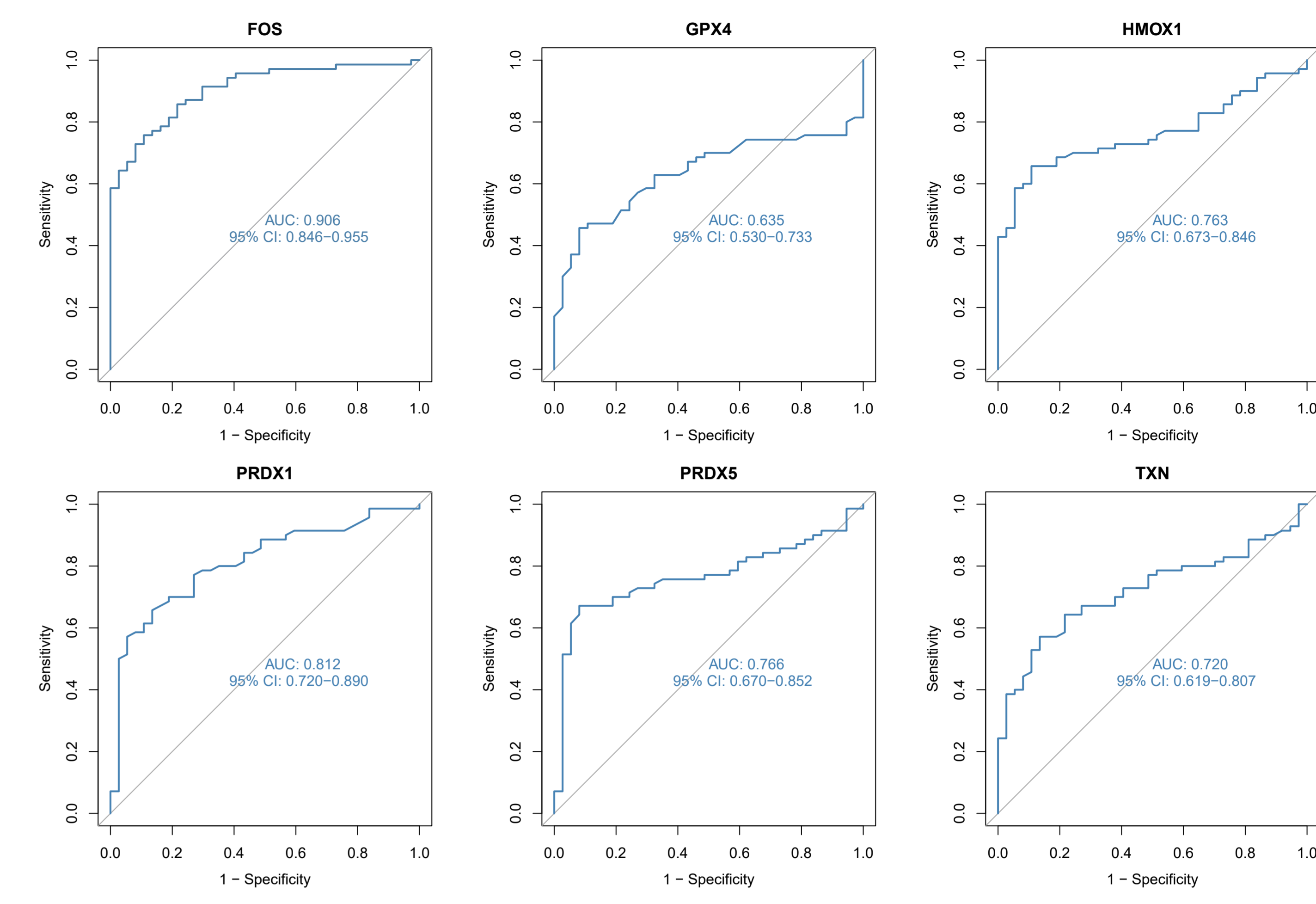

GSE36376

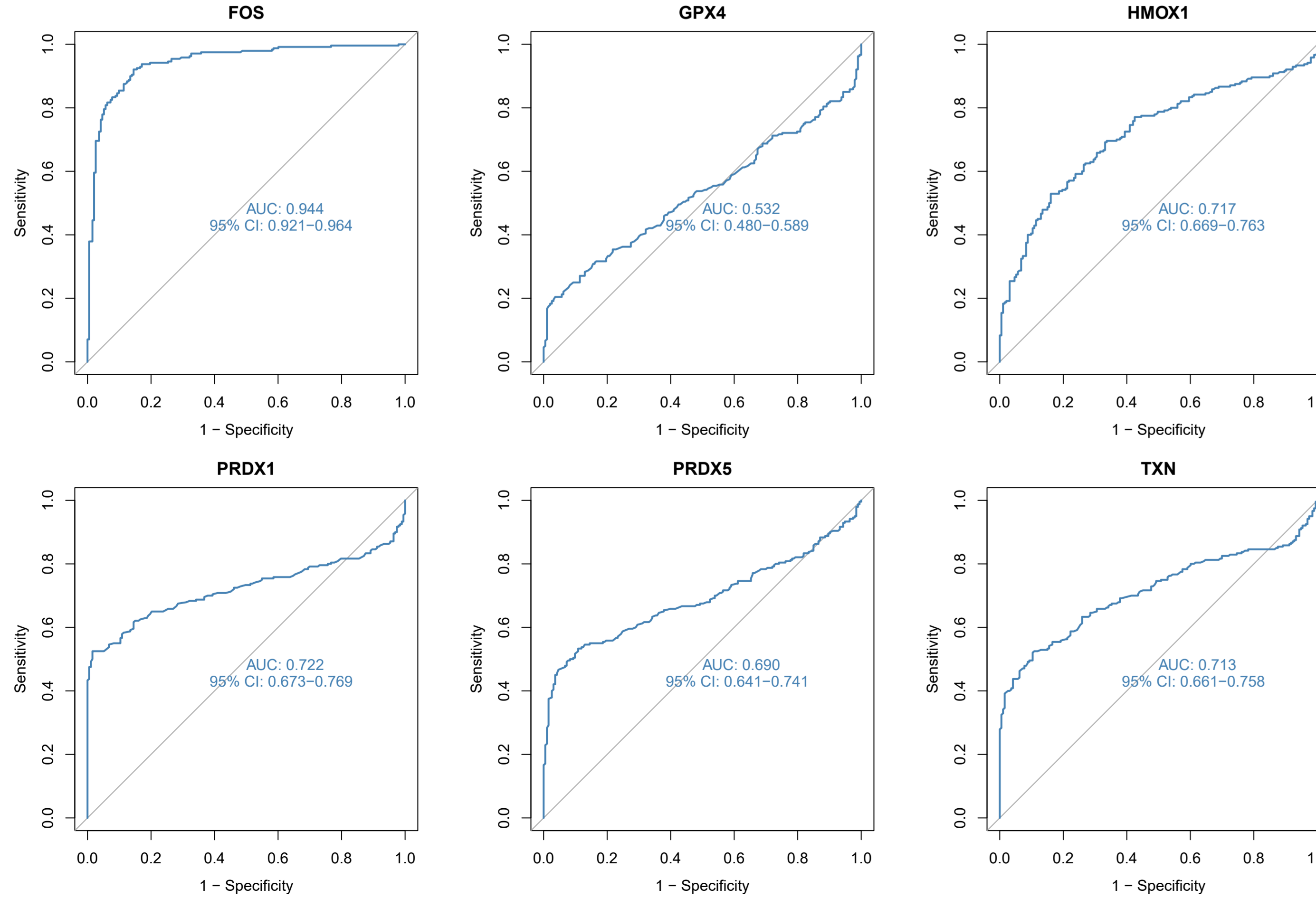

GSE69715

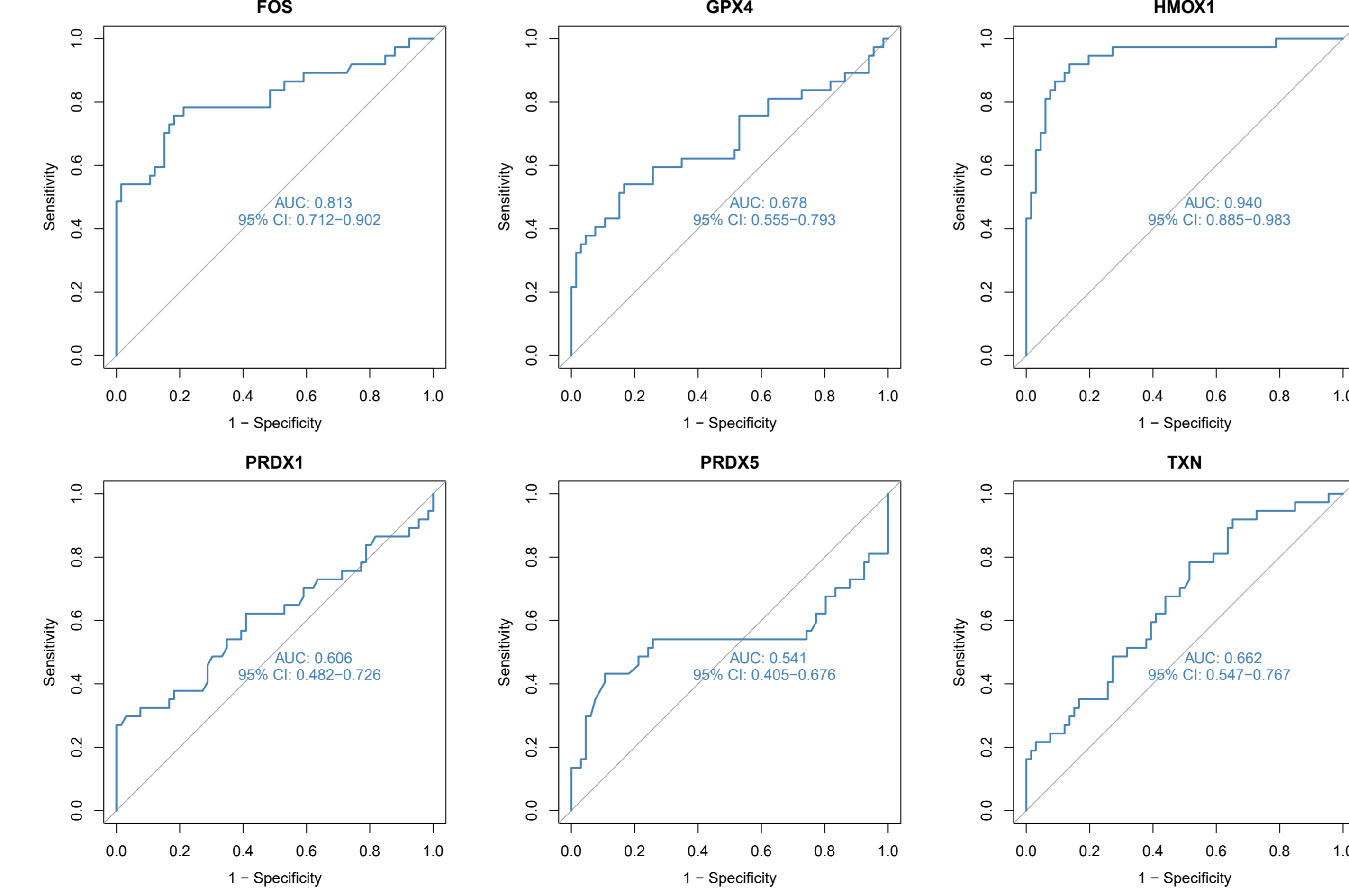

GSE45267

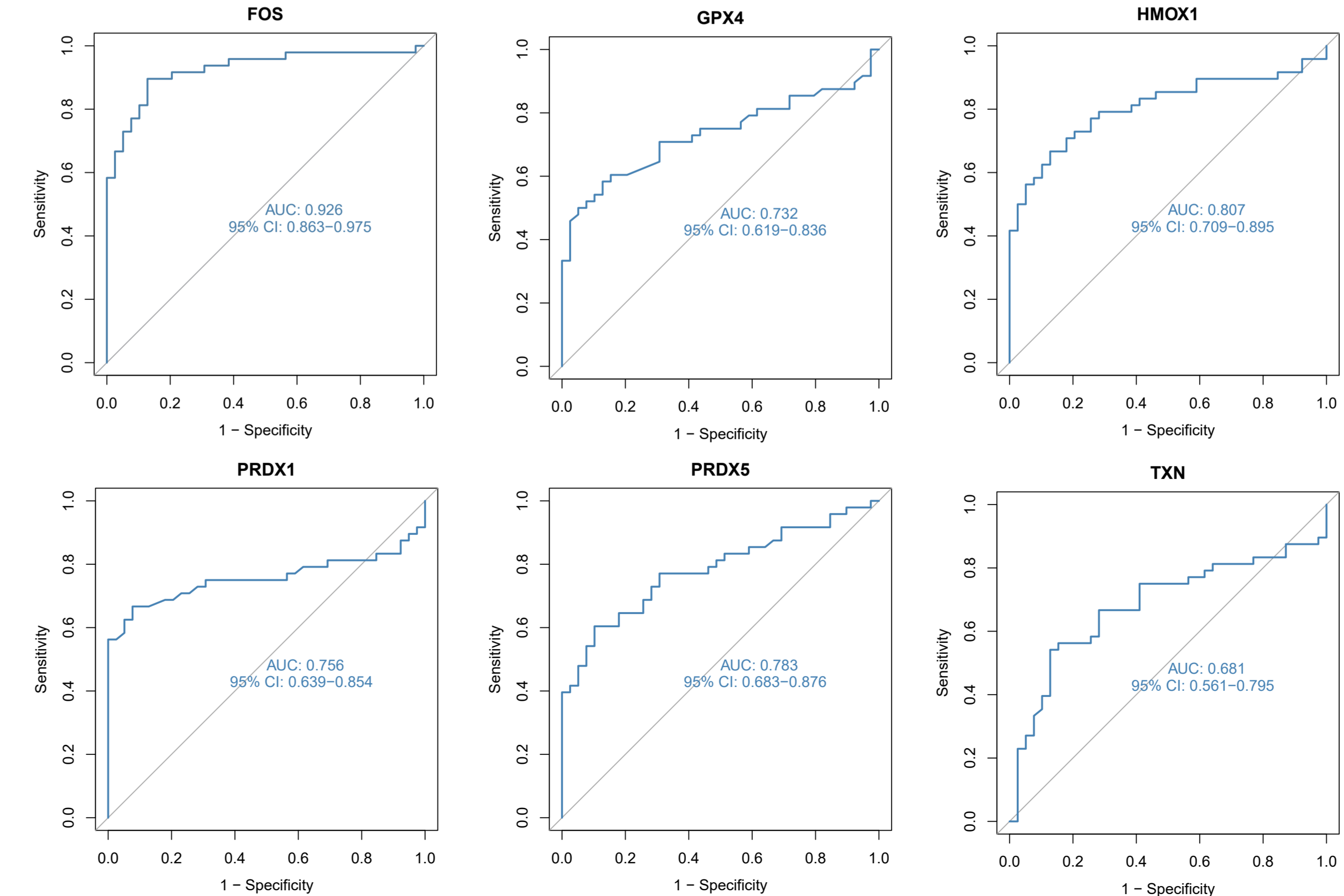

Training cohort (GTEx-TCGA)

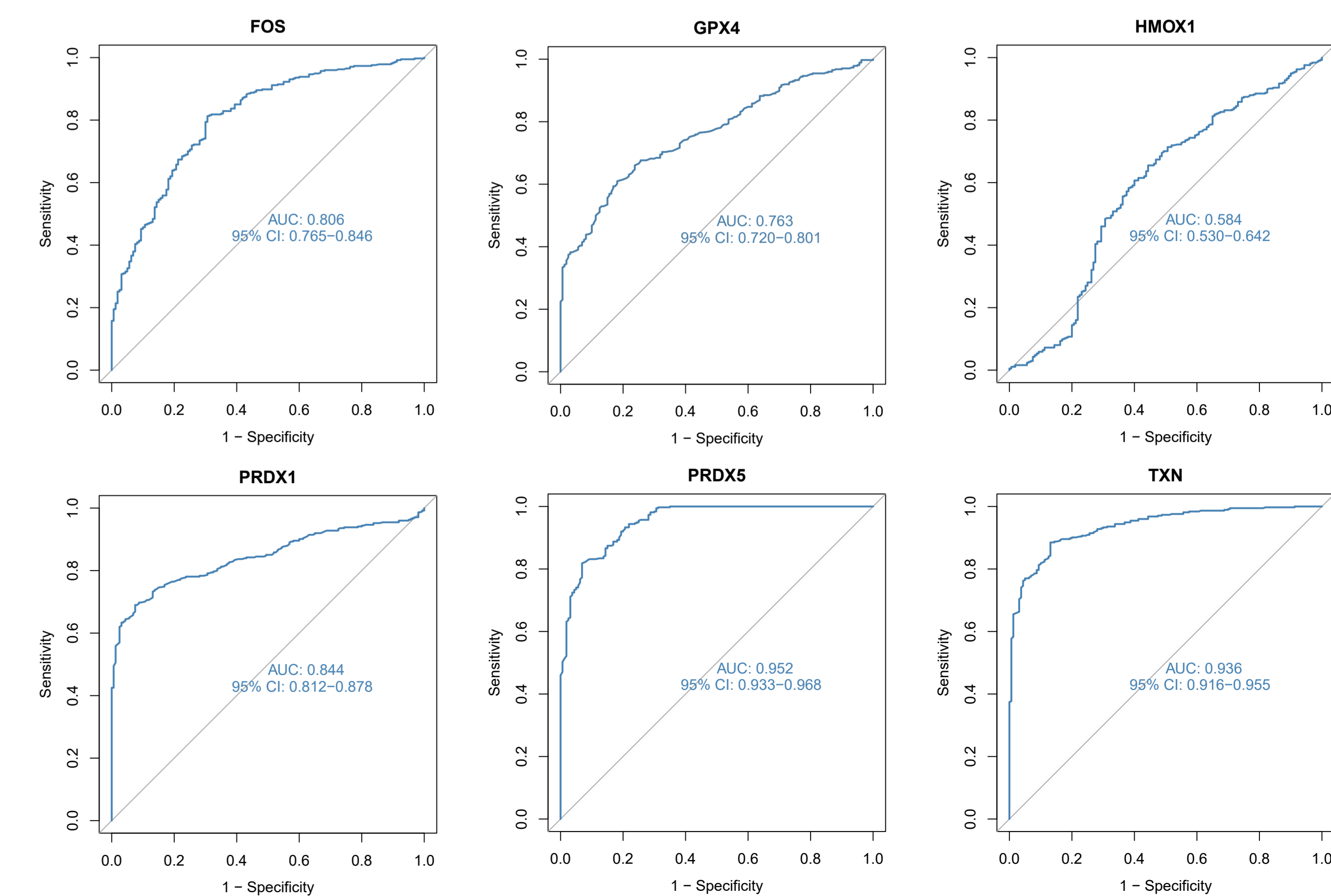

Supplement: Supplementary file 8 [file Image8.PDF]

**A**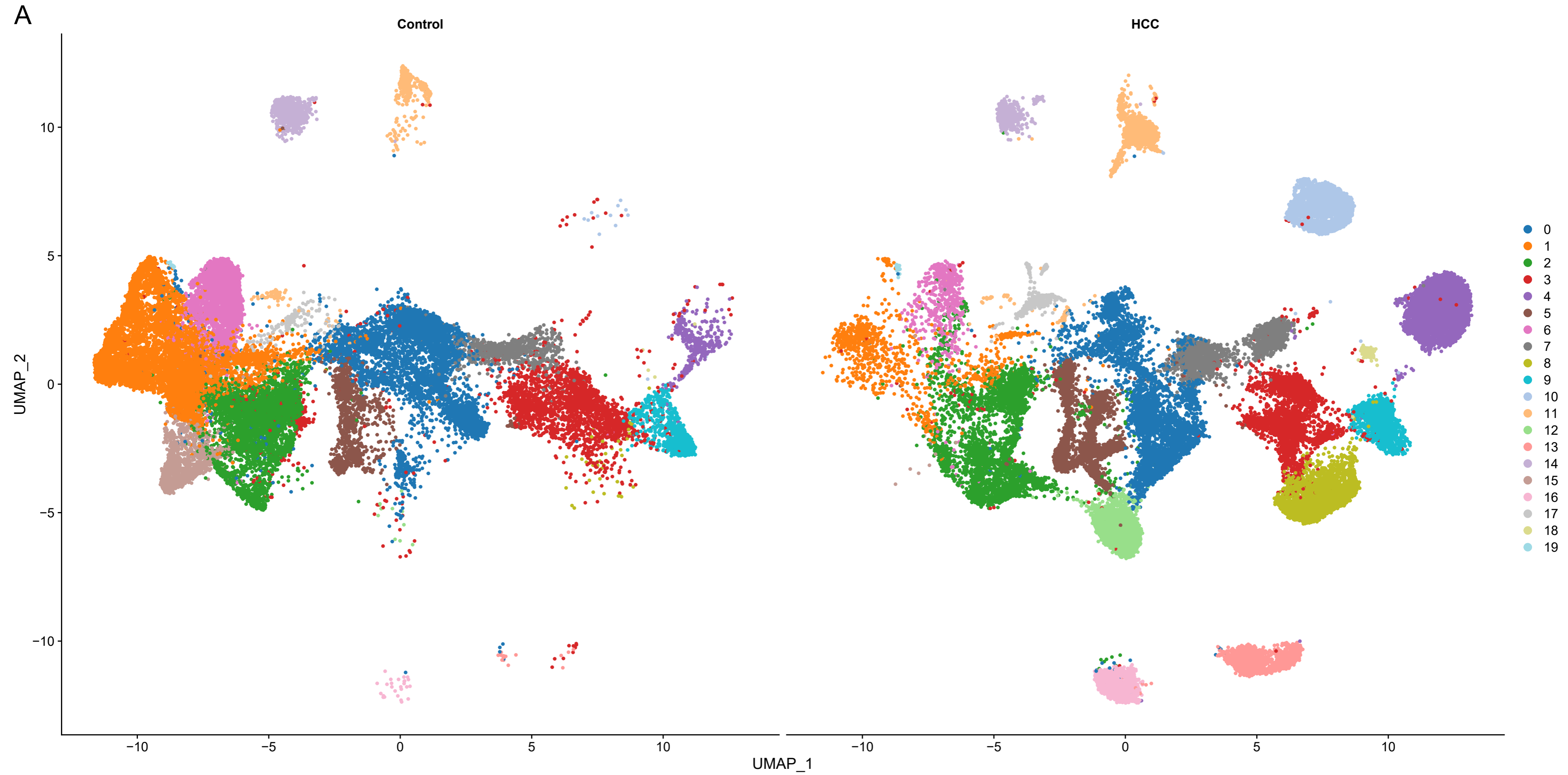**B**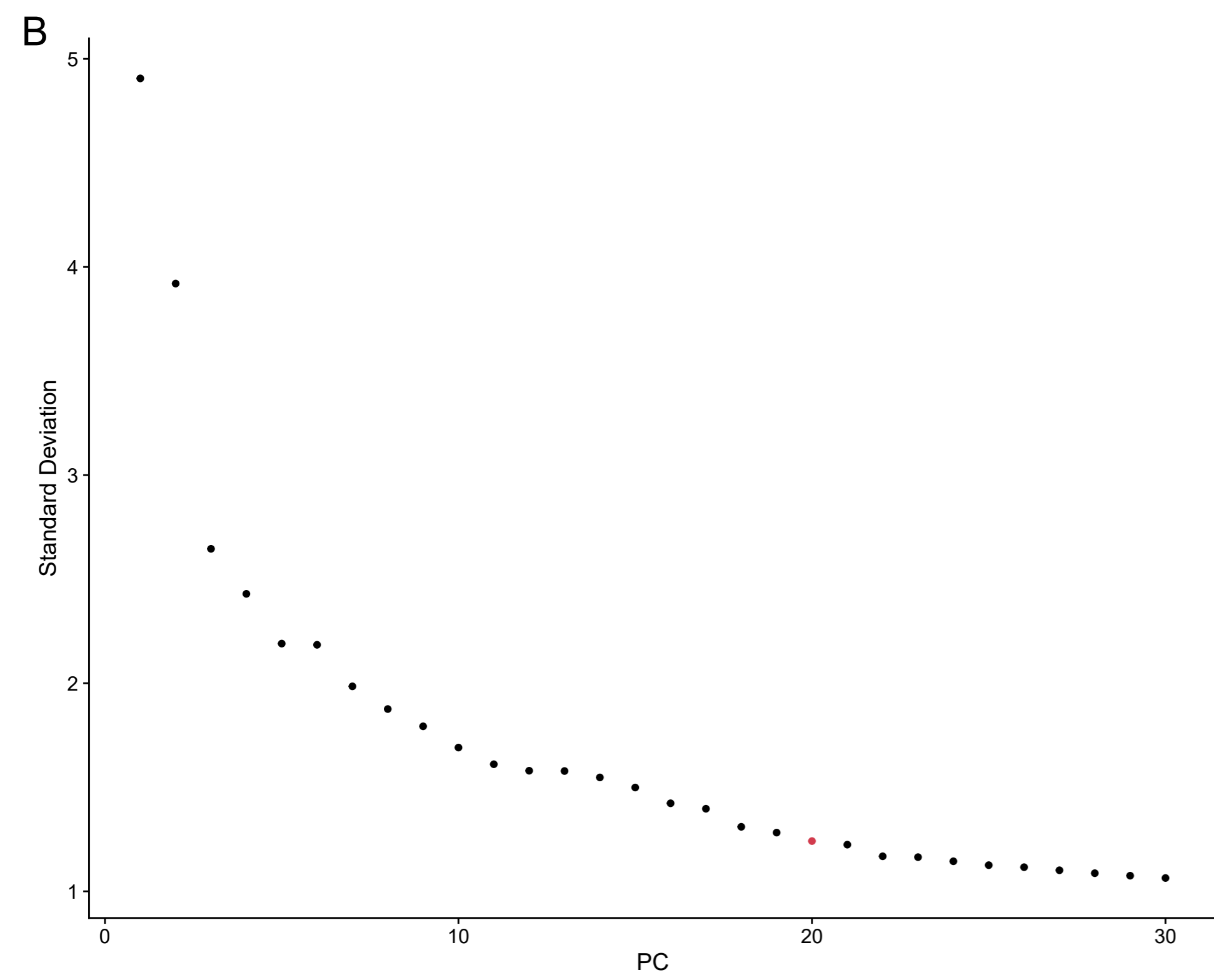

Supplement: Supplementary file 10 [file Image2.PDF]

**A**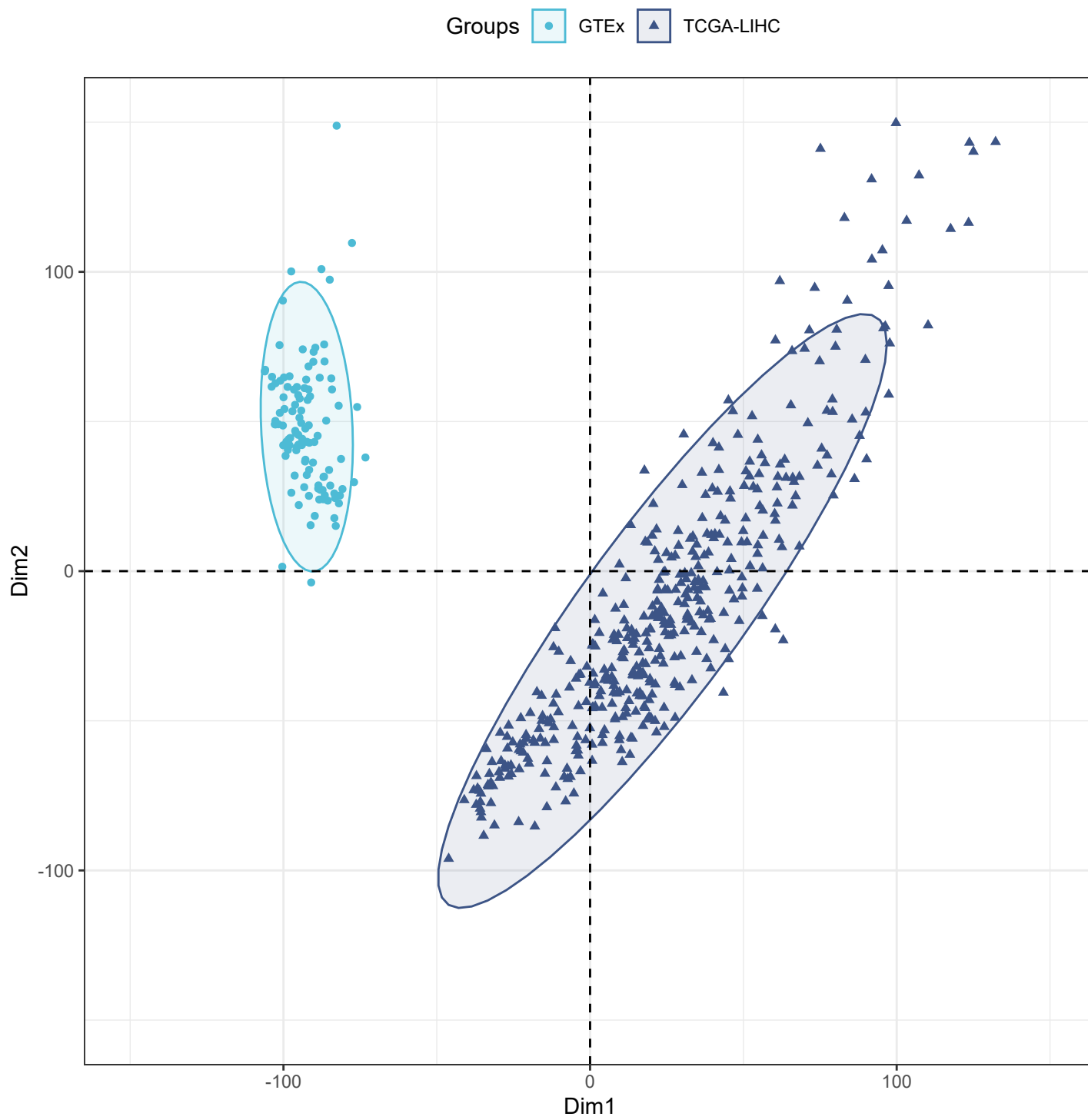**B**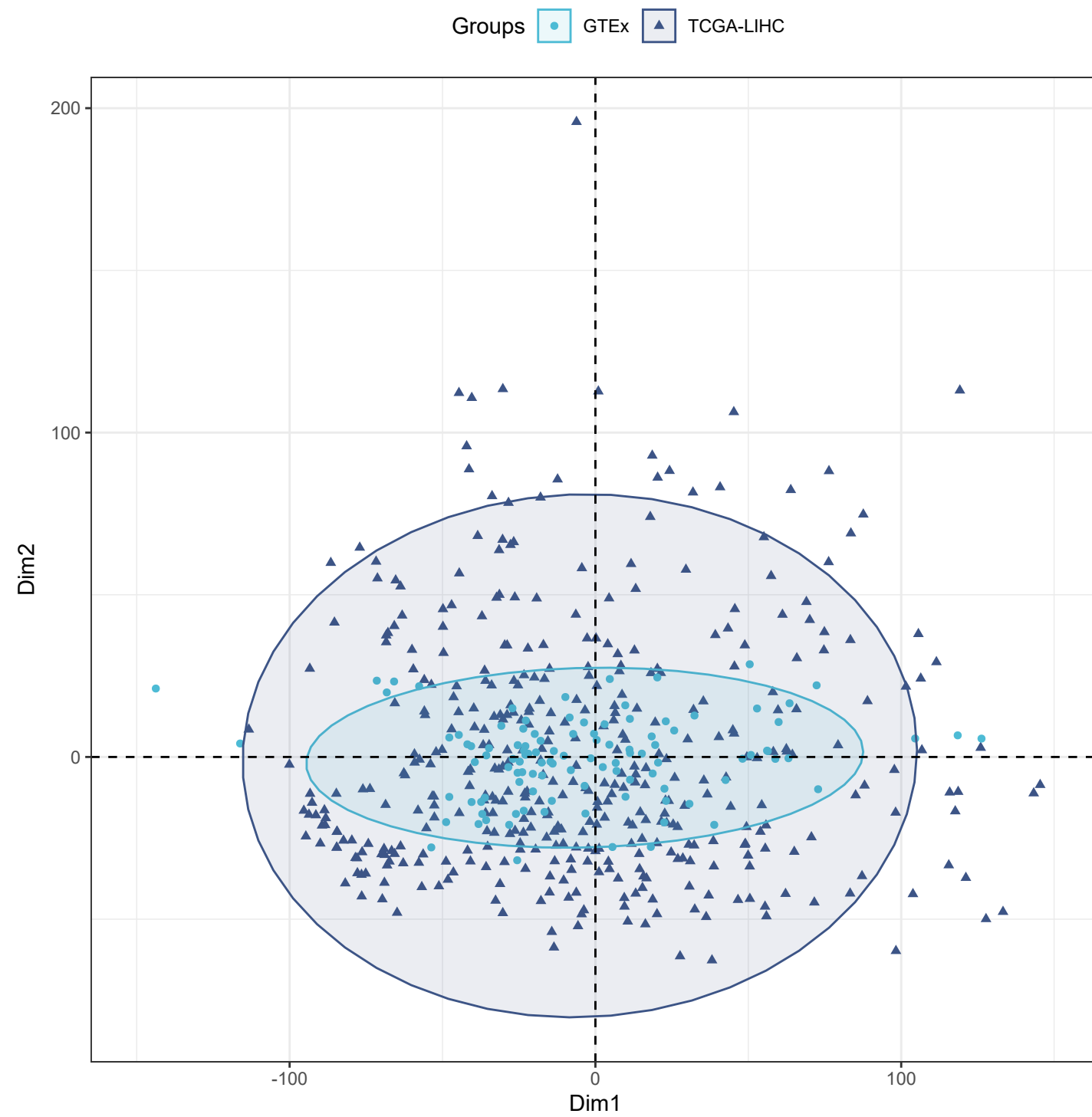

Supplement: Supplementary file 11 [file Image3.PDF]

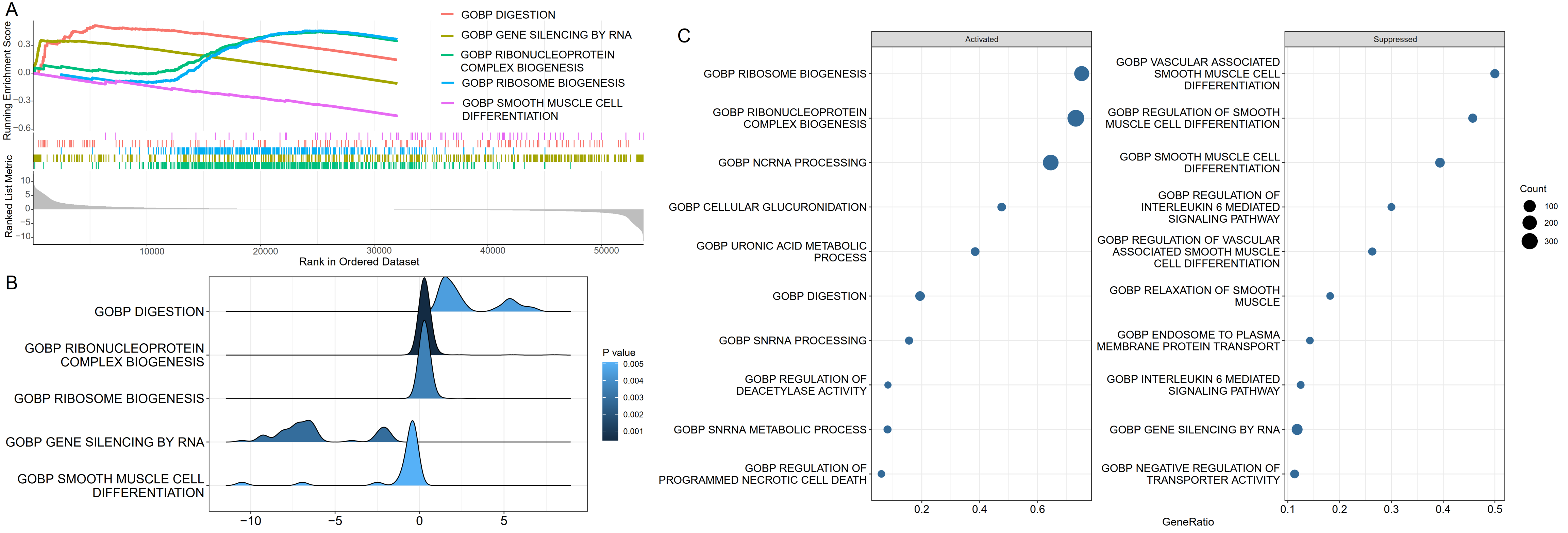

Supplement: Supplementary file 14 [file Image11.PDF]

A

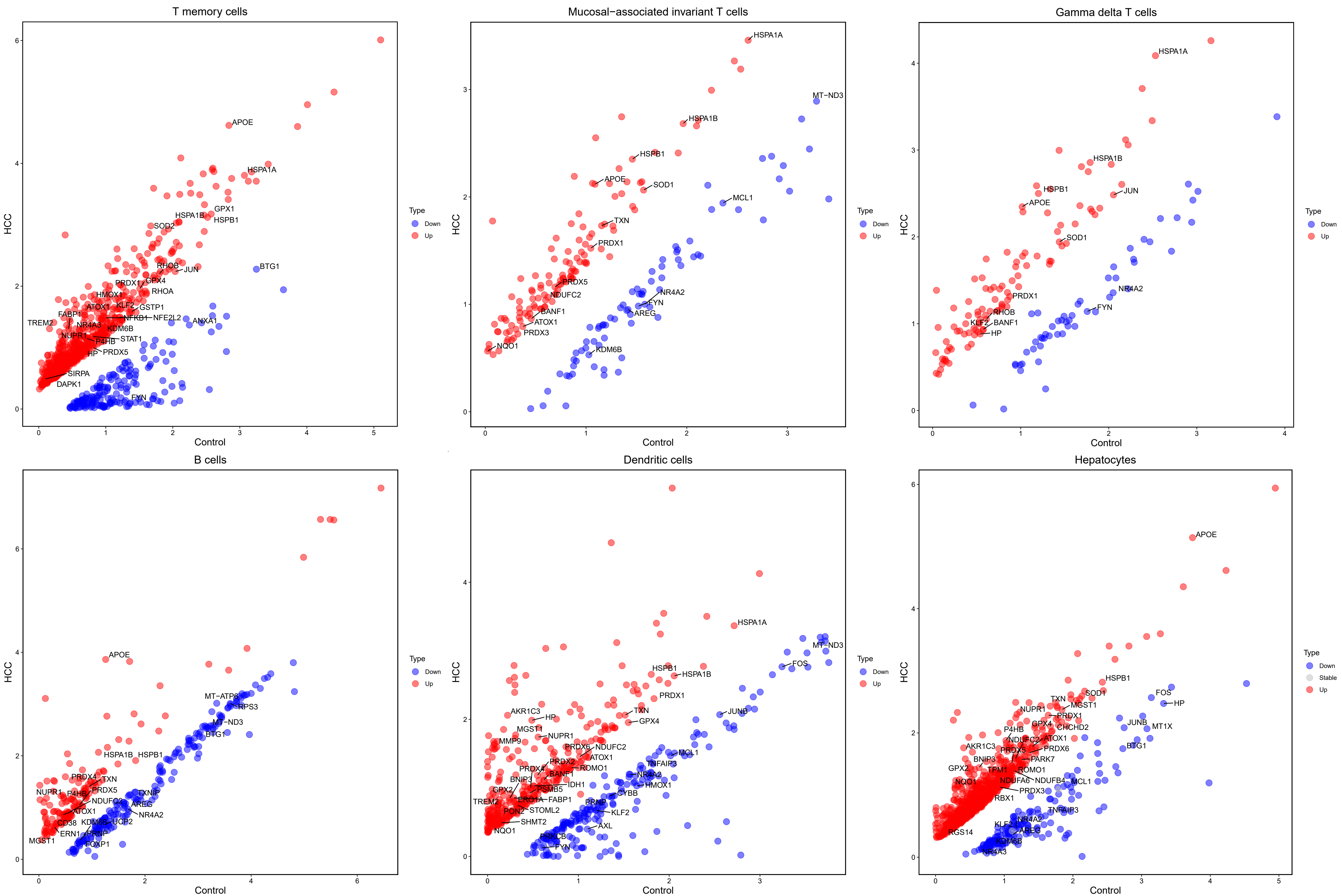

B

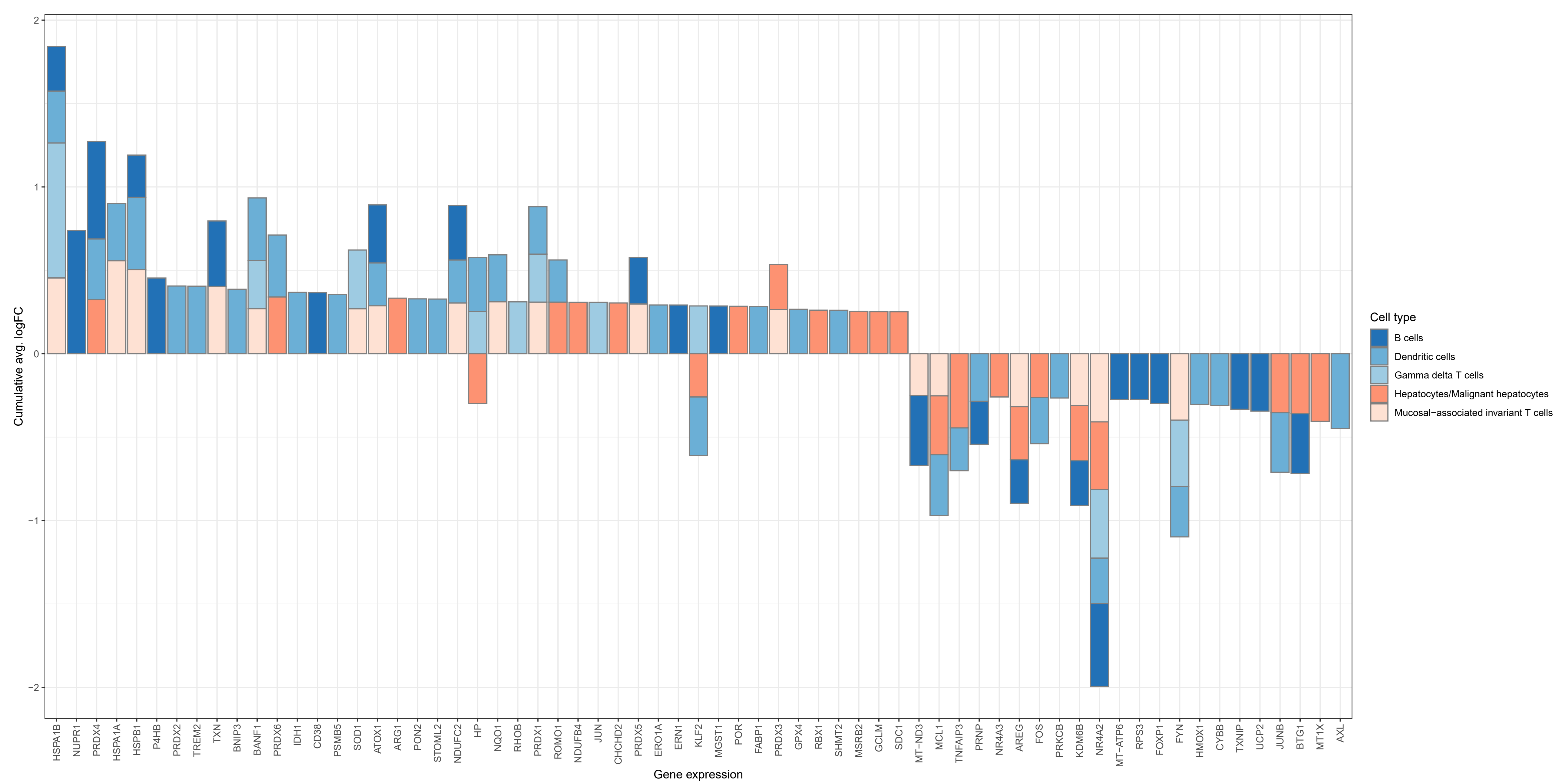

Supplement: Supplementary file 15 [file Image7.PDF]

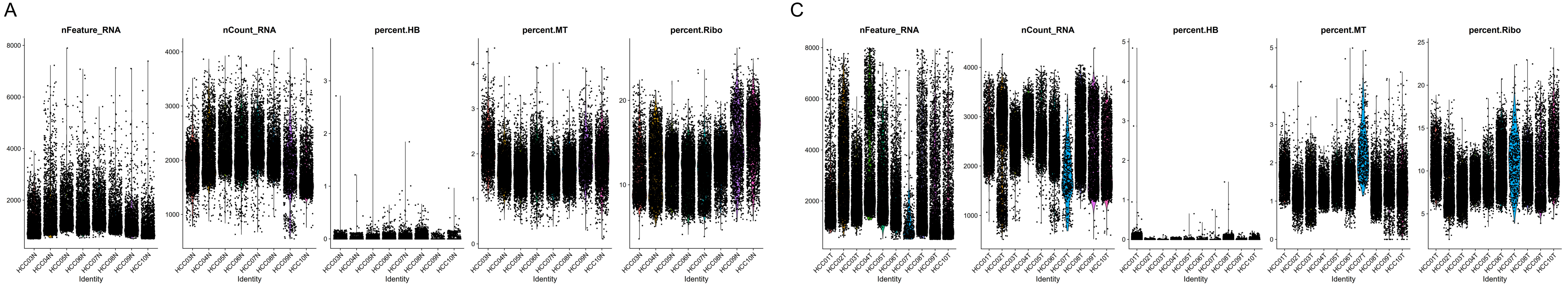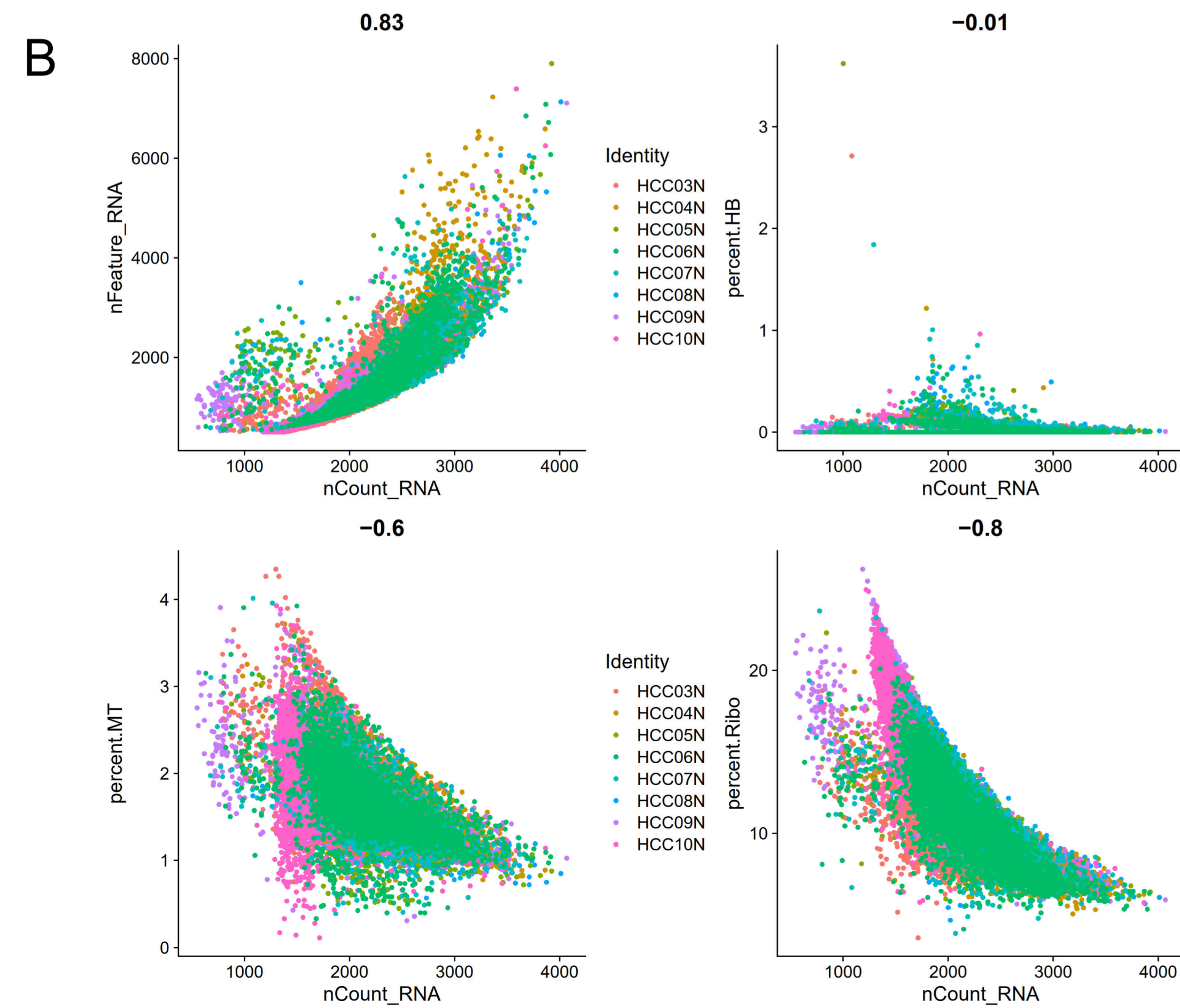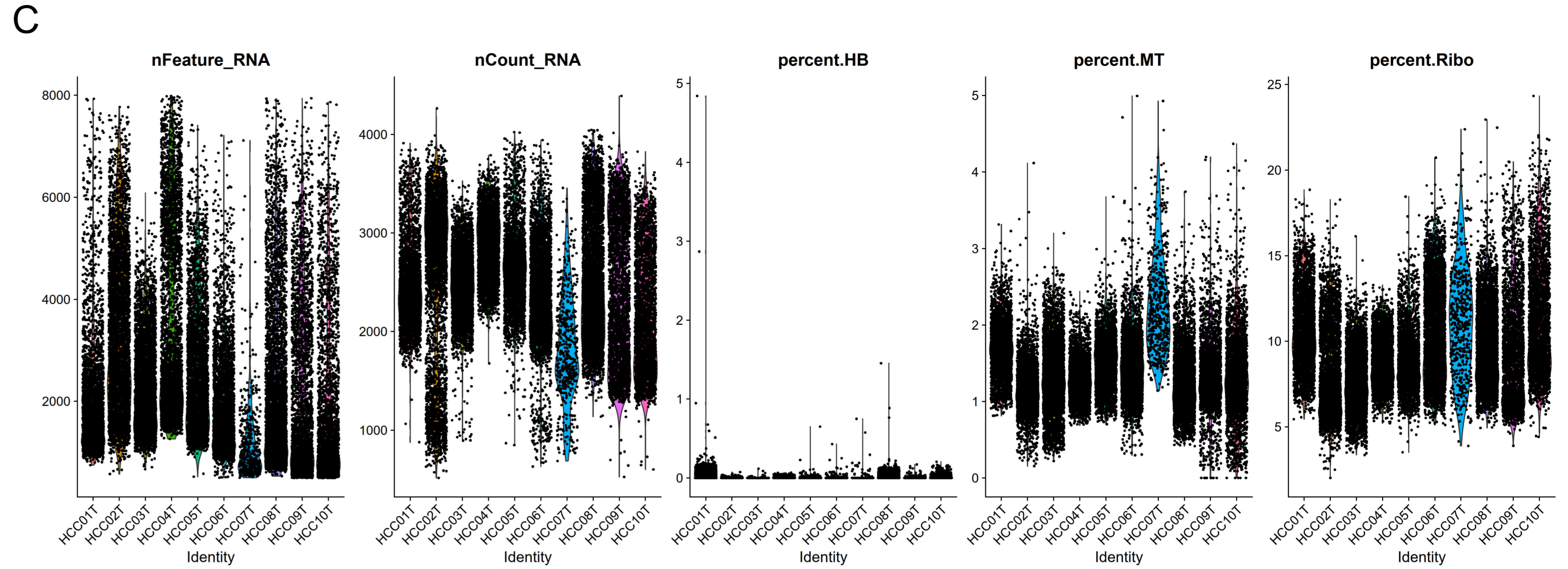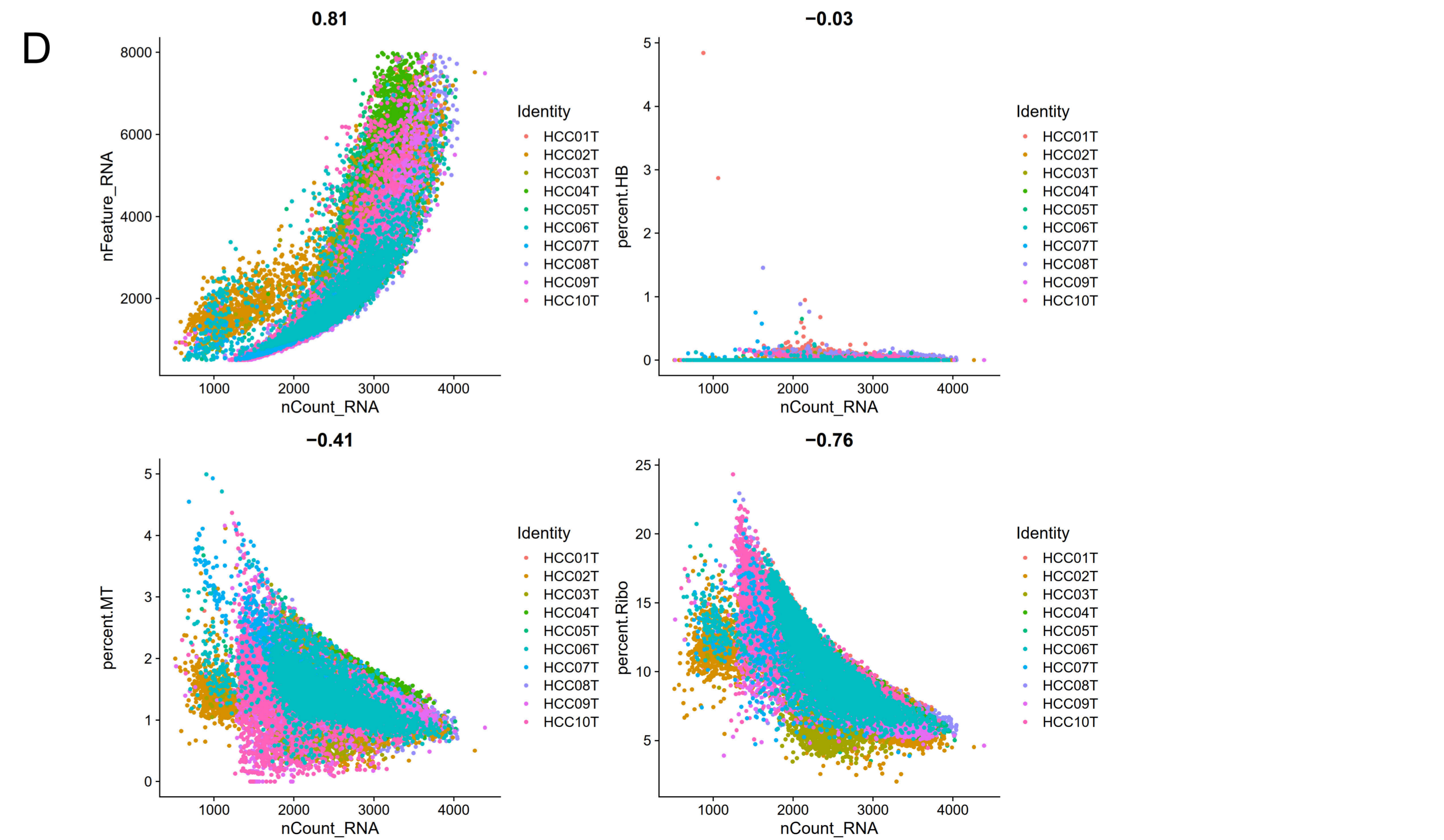

Supplement: Supplementary file 18 [file Image1.PDF]
