# Supplementary material for: Single cell and bulk transcriptome analysis identified oxidative stress response-related features of Hepatocellular Carcinoma
Source: Front Cell Dev Biol. 2023 Sep 28;11:1191074. doi: 10.3389/fcell.2023.1191074 (PMC10568628; doi:10.3389/fcell.2023.1191074)
Supplement: Supplementary file 4 [file Image5.PDF]

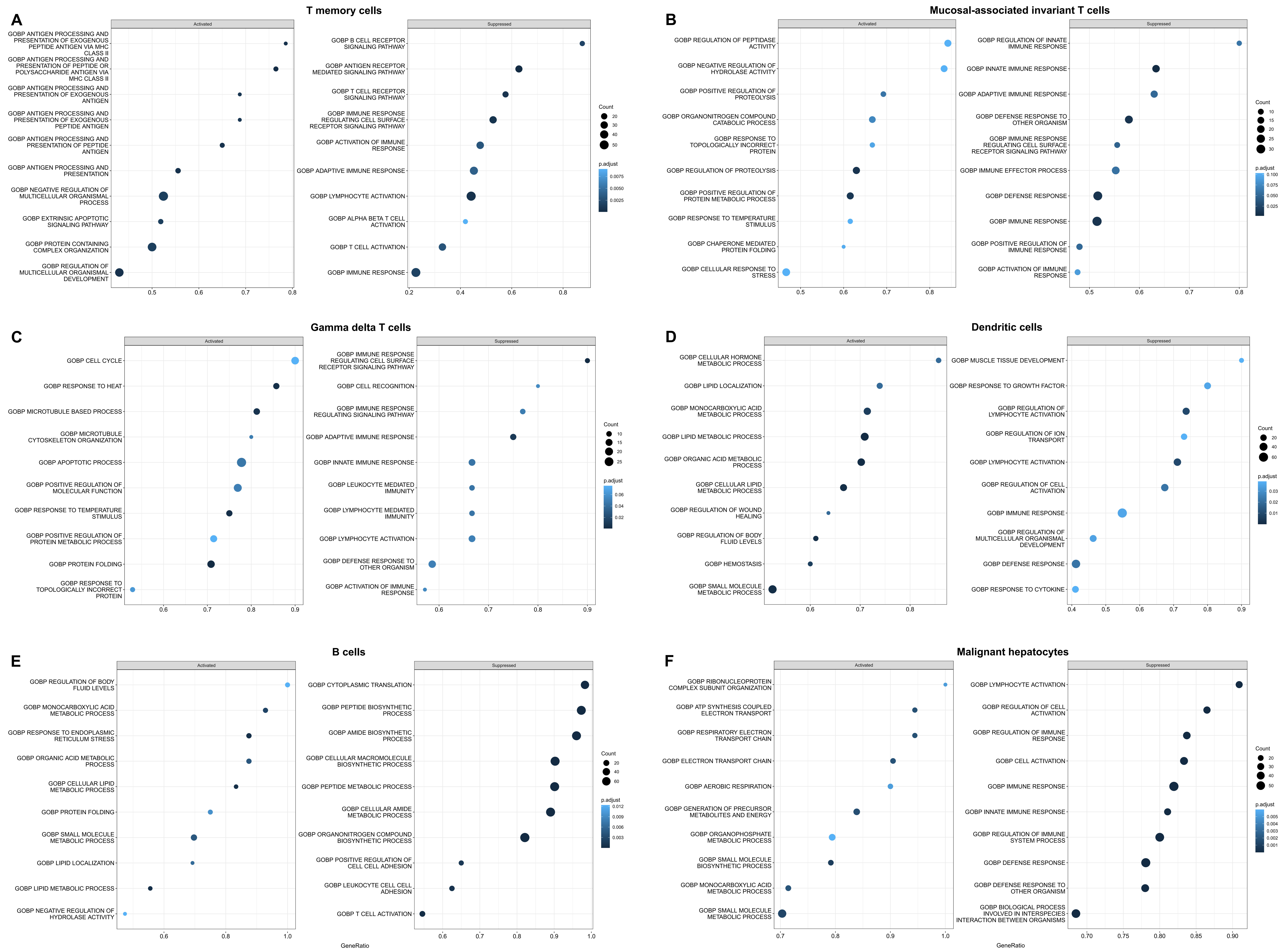

A
T memory cells
Activated
Suppressed
GOBP ANTIGEN PROCESSING AND PRESENTATION OF EXOGENOUS PEPTIDE ANTIGEN VIA MHC CLASS II
GOBP ANTIGEN PROCESSING AND PRESENTATION OF PEPTIDE OR POLYSACCHARIDE ANTIGEN VIA MHC CLASS II
GOBP ANTIGEN PROCESSING AND PRESENTATION OF EXOGENOUS ANTIGEN
GOBP ANTIGEN PROCESSING AND PRESENTATION OF EXOGENOUS PEPTIDE ANTIGEN
GOBP ANTIGEN PROCESSING AND PRESENTATION OF PEPTIDE ANTIGEN
GOBP ANTIGEN PROCESSING AND PRESENTATION
GOBP NEGATIVE REGULATION OF MULTICELLULAR ORGANISMAL PROCESS
GOBP EXTRINSIC APOPTOTIC SIGNALING PATHWAY
GOBP PROTEIN CONTAINING COMPLEX ORGANIZATION
GOBP REGULATION OF MULTICELLULAR ORGANISMAL DEVELOPMENT
GOBP B CELL RECEPTOR SIGNALING PATHWAY
GOBP ANTIGEN RECEPTOR MEDIATED SIGNALING PATHWAY
GOBP T CELL RECEPTOR SIGNALING PATHWAY
GOBP IMMUNE RESPONSE REGULATING CELL SURFACE RECEPTOR SIGNALING PATHWAY
GOBP ACTIVATION OF IMMUNE RESPONSE
GOBP ADAPTIVE IMMUNE RESPONSE
GOBP LYMPHOCYTE ACTIVATION
GOBP ALPHA BETA T CELL ACTIVATION
GOBP T CELL ACTIVATION
GOBP IMMUNE RESPONSE
0.5 0.6 0.7 0.8
0.2 0.4 0.6 0.8
Count
20
30
40
50
p.adjust
0.0075
0.0050
0.0025
B
Mucosal-associated invariant T cells
Activated
Suppressed
GOBP REGULATION OF PEPTIDASE ACTIVITY
GOBP NEGATIVE REGULATION OF HYDROLASE ACTIVITY
GOBP POSITIVE REGULATION OF PROTEOLYSIS
GOBP ORGANONITROGEN COMPOUND CATABOLIC PROCESS
GOBP RESPONSE TO TOPOLOGICALLY INCORRECT PROTEIN
GOBP REGULATION OF PROTEOLYSIS
GOBP POSITIVE REGULATION OF PROTEIN METABOLIC PROCESS
GOBP RESPONSE TO TEMPERATURE STIMULUS
GOBP CHAPERONE MEDIATED PROTEIN FOLDING
GOBP CELLULAR RESPONSE TO STRESS
GOBP REGULATION OF INNATE IMMUNE RESPONSE
GOBP INNATE IMMUNE RESPONSE
GOBP ADAPTIVE IMMUNE RESPONSE
GOBP DEFENSE RESPONSE TO OTHER ORGANISM
GOBP IMMUNE RESPONSE REGULATING CELL SURFACE RECEPTOR SIGNALING PATHWAY
GOBP IMMUNE EFFECTOR PROCESS
GOBP DEFENSE RESPONSE
GOBP IMMUNE RESPONSE
GOBP POSITIVE REGULATION OF IMMUNE RESPONSE
GOBP ACTIVATION OF IMMUNE RESPONSE
0.5 0.6 0.7 0.8
0.5 0.6 0.7 0.8
Count
10
15
20
25
30
p.adjust
0.100
0.075
0.050
0.025
C
Gamma delta T cells
Activated
Suppressed
GOBP CELL CYCLE
GOBP RESPONSE TO HEAT
GOBP MICROTUBULE BASED PROCESS
GOBP MICROTUBULE CYTOSKELETON ORGANIZATION
GOBP APOPTOTIC PROCESS
GOBP POSITIVE REGULATION OF MOLECULAR FUNCTION
GOBP RESPONSE TO TEMPERATURE STIMULUS
GOBP POSITIVE REGULATION OF PROTEIN METABOLIC PROCESS
GOBP PROTEIN FOLDING
GOBP RESPONSE TO TOPOLOGICALLY INCORRECT PROTEIN
GOBP IMMUNE RESPONSE REGULATING CELL SURFACE RECEPTOR SIGNALING PATHWAY
GOBP CELL RECOGNITION
GOBP IMMUNE RESPONSE REGULATING SIGNALING PATHWAY
GOBP ADAPTIVE IMMUNE RESPONSE
GOBP INNATE IMMUNE RESPONSE
GOBP LEUKOCYTE MEDIATED IMMUNITY
GOBP LYMPHOCYTE MEDIATED IMMUNITY
GOBP LYMPHOCYTE ACTIVATION
GOBP DEFENSE RESPONSE TO OTHER ORGANISM
GOBP ACTIVATION OF IMMUNE RESPONSE
0.6 0.7 0.8 0.9
0.6 0.7 0.8 0.9
Count
10
15
20
25
p.adjust
0.06
0.04
0.02
D
Dendritic cells
Activated
Suppressed
GOBP CELLULAR HORMONE METABOLIC PROCESS
GOBP LIPID LOCALIZATION
GOBP MONOCARBOXYLIC ACID METABOLIC PROCESS
GOBP LIPID METABOLIC PROCESS
GOBP ORGANIC ACID METABOLIC PROCESS
GOBP CELLULAR LIPID METABOLIC PROCESS
GOBP REGULATION OF WOUND HEALING
GOBP REGULATION OF BODY FLUID LEVELS
GOBP HEMOSTASIS
GOBP SMALL MOLECULE METABOLIC PROCESS
GOBP MUSCLE TISSUE DEVELOPMENT
GOBP RESPONSE TO GROWTH FACTOR
GOBP REGULATION OF LYMPHOCYTE ACTIVATION
GOBP REGULATION OF ION TRANSPORT
GOBP LYMPHOCYTE ACTIVATION
GOBP REGULATION OF CELL ACTIVATION
GOBP IMMUNE RESPONSE
GOBP REGULATION OF MULTICELLULAR ORGANISMAL DEVELOPMENT
GOBP DEFENSE RESPONSE
GOBP RESPONSE TO CYTOKINE
0.6 0.7 0.8
0.4 0.5 0.6 0.7 0.8 0.9
Count
20
40
60
p.adjust
0.03
0.02
0.01
E
B cells
Activated
Suppressed
GOBP REGULATION OF BODY FLUID LEVELS
GOBP MONOCARBOXYLIC ACID METABOLIC PROCESS
GOBP RESPONSE TO ENDOPLASMIC RETICULUM STRESS
GOBP ORGANIC ACID METABOLIC PROCESS
GOBP CELLULAR LIPID METABOLIC PROCESS
GOBP PROTEIN FOLDING
GOBP SMALL MOLECULE METABOLIC PROCESS
GOBP LIPID LOCALIZATION
GOBP LIPID METABOLIC PROCESS
GOBP NEGATIVE REGULATION OF HYDROLASE ACTIVITY
GOBP CYTOPLASMIC TRANSLATION
GOBP PEPTIDE BIOSYNTHETIC PROCESS
GOBP AMIDE BIOSYNTHETIC PROCESS
GOBP CELLULAR MACROMOLECULE BIOSYNTHETIC PROCESS
GOBP PEPTIDE METABOLIC PROCESS
GOBP CELLULAR AMIDE METABOLIC PROCESS
GOBP ORGANONITROGEN COMPOUND BIOSYNTHETIC PROCESS
GOBP POSITIVE REGULATION OF CELL CELL ADHESION
GOBP LEUKOCYTE CELL CELL ADHESION
GOBP T CELL ACTIVATION
0.6 0.8 1.0
0.6 0.7 0.8 0.9 1.0
GeneRatio
Count
20
40
60
p.adjust
0.012
0.009
0.006
0.003
F
Malignant hepatocytes
Activated
Suppressed
GOBP RIBONUCLEOPROTEIN COMPLEX SUBUNIT ORGANIZATION
GOBP ATP SYNTHESIS COUPLED ELECTRON TRANSPORT
GOBP RESPIRATORY ELECTRON TRANSPORT CHAIN
GOBP ELECTRON TRANSPORT CHAIN
GOBP AEROBIC RESPIRATION
GOBP GENERATION OF PRECURSOR METABOLITES AND ENERGY
GOBP ORGANOPHOSPHATE METABOLIC PROCESS
GOBP SMALL MOLECULE BIOSYNTHETIC PROCESS
GOBP MONOCARBOXYLIC ACID METABOLIC PROCESS
GOBP SMALL MOLECULE METABOLIC PROCESS
GOBP LYMPHOCYTE ACTIVATION
GOBP REGULATION OF CELL ACTIVATION
GOBP REGULATION OF IMMUNE RESPONSE
GOBP CELL ACTIVATION
GOBP IMMUNE RESPONSE
GOBP INNATE IMMUNE RESPONSE
GOBP REGULATION OF IMMUNE SYSTEM PROCESS
GOBP DEFENSE RESPONSE
GOBP DEFENSE RESPONSE TO OTHER ORGANISM
GOBP BIOLOGICAL PROCESS INVOLVED IN INTERSPECIES INTERACTION BETWEEN ORGANISMS
0.7 0.8 0.9 1.0
0.70 0.75 0.80 0.85 0.90
GeneRatio
Count
20
30
40
50
p.adjust
0.005
0.004
0.003
0.002
0.001
