# Supplementary material for: Single cell and bulk transcriptome analysis identified oxidative stress response-related features of Hepatocellular Carcinoma
Source: Front Cell Dev Biol. 2023 Sep 28;11:1191074. doi: 10.3389/fcell.2023.1191074 (PMC10568628; doi:10.3389/fcell.2023.1191074)
Supplement: Supplementary file 5 [file Image9.PDF]

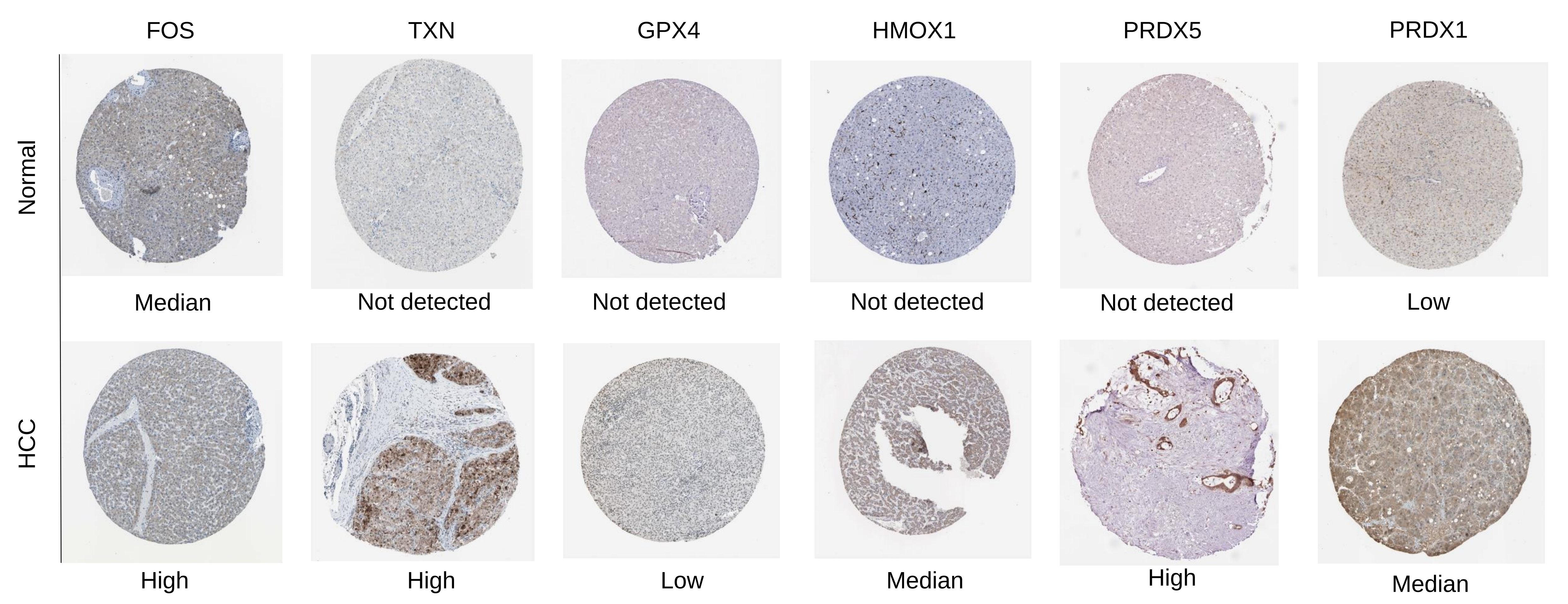

FOS
TXN
GPX4
HMOX1
PRDX5
PRDX1
Normal
HCC
Median
Not detected
Not detected
Not detected
Not detected
Low
High
High
Low
Median
High
Median
