# Supplementary material for: Single cell and bulk transcriptome analysis identified oxidative stress response-related features of Hepatocellular Carcinoma
Source: Front Cell Dev Biol. 2023 Sep 28;11:1191074. doi: 10.3389/fcell.2023.1191074 (PMC10568628; doi:10.3389/fcell.2023.1191074)
Supplement: Supplementary file 9 [file Image4.PDF]

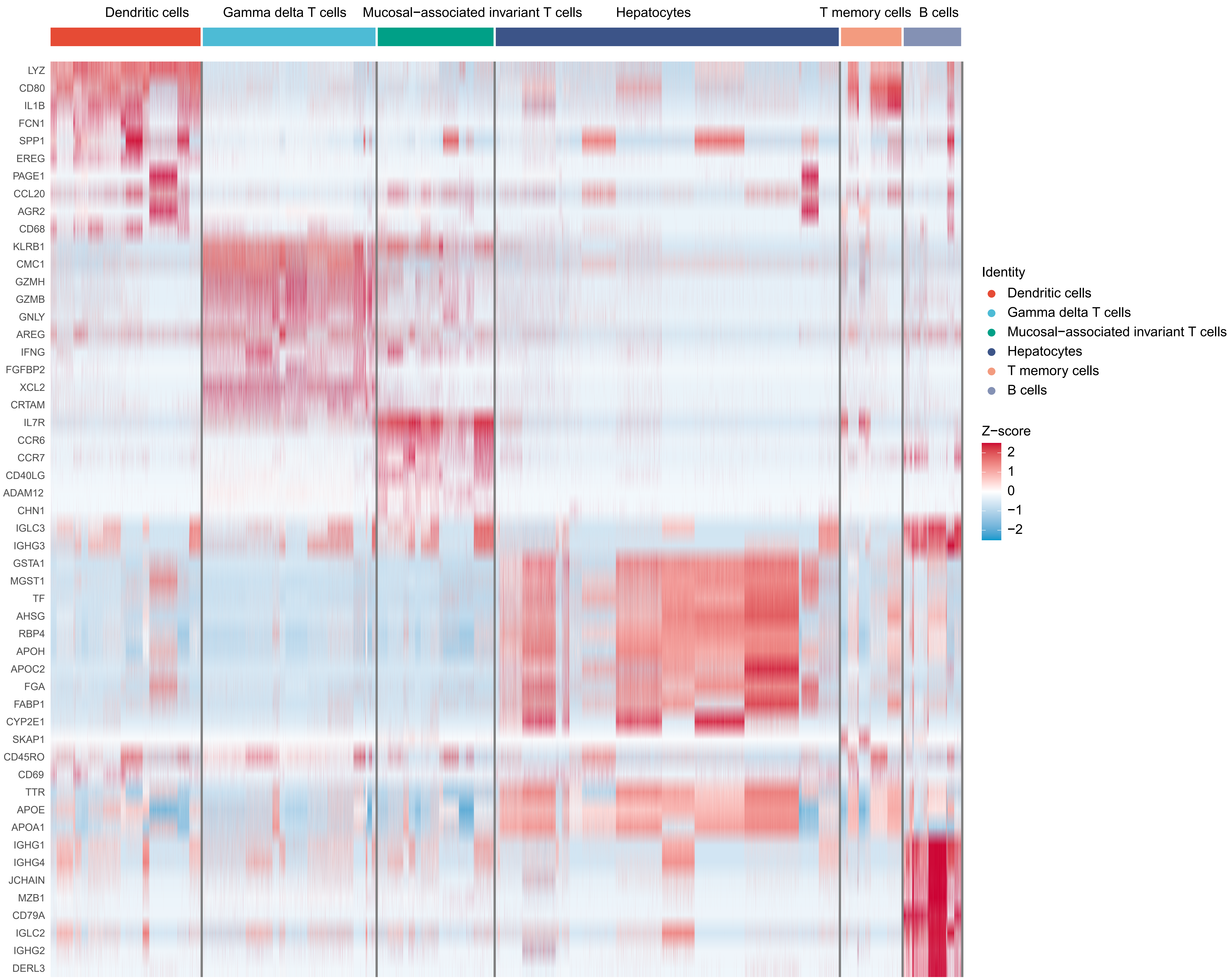

Dendritic cells
Gamma delta T cells
Mucosal−associated invariant T cells
Hepatocytes
T memory cells
B cells
LYZ
CD80
IL1B
FCN1
SPP1
EREG
PAGE1
CCL20
AGR2
CD68
KLRB1
CMC1
GZMH
GZMB
GNLY
AREG
IFNG
FGFBP2
XCL2
CRTAM
IL7R
CCR6
CCR7
CD40LG
ADAM12
CHN1
IGLC3
IGHG3
GSTA1
MGST1
TF
AHSG
RBP4
APOH
APOC2
FGA
FABP1
CYP2E1
SKAP1
CD45RO
CD69
TTR
APOE
APOA1
IGHG1
IGHG4
JCHAIN
MZB1
CD79A
IGLC2
IGHG2
DERL3
Identity
Dendritic cells
Gamma delta T cells
Mucosal−associated invariant T cells
Hepatocytes
T memory cells
B cells
Z−score
2
1
0
−1
−2
