# Supplementary material for: Single cell and bulk transcriptome analysis identified oxidative stress response-related features of Hepatocellular Carcinoma
Source: Front Cell Dev Biol. 2023 Sep 28;11:1191074. doi: 10.3389/fcell.2023.1191074 (PMC10568628; doi:10.3389/fcell.2023.1191074)
Supplement: Supplementary file 17 [file DataSheet1.DOCX]

**Supplementary** **materials information**

**Supplementary Tables:**

Supplementary Table 1. Clinical and sample information of datasets used in our study.

Supplementary Table 2. Brief description of genes involved in oxidative stress response identified from Molecular Signatures database.

Supplementary Table 3. Differentially expressed oxidative stress-related genes of each cell cluster between non-tumor samples and HCC samples.

Supplementary Table 4. Top six gene signature variables in each machine learning model.

Supplementary Table 5. The outcomes of univariate and multivariate cox regression analyses in TCGA-LIHC and HCCDB18 datasets.

**Supplementary Figures:**

Supplementary Figure 1. Quality control of the scRNA-seq data. (A, B) Number of genes and relative hemoglobin, mitochondrial, and ribosomal transcript abundance from non-tumor liver cells. (C, D) The number of genes and relative hemoglobin, mitochondrial, and ribosomal transcript abundance from HCC cells.

Supplementary Figure 2. UMAP visualization results of single-cell data after dimension reduction by PCA. (A) A plot of UMAP subgroups after PCA dimensionality reduction for the non-tumor group versus the HCC group. (B) The PC was chosen from elbow plot of PCA analysis.

Supplementary Figure 3. PCA dimension reduction views of two datasets before (A) and after (B) removal of batch effects.

Supplementary Figure 4. Marker genes heatmap for cell groups.

Supplementary Figure 5. Biology process enrichment analysis of cell clusters in HCC samples. The bubble charts of activated and suppressed biology functions in (A) T memory cells, (B) Mucosal-associated invariant T cells, (C) Gamma delta T cells, (D) Dendritic cells, (E) B cells, and (F) Malignant hepatocytes.

Supplementary Figure 6. An independent map of intercellular communication.

Supplementary Figure 7. The oxidative stress-related DEGs in HCC. (A) Volcano plots of differentially expressed genes of cell clusters between non-tumor tissues and HCC tissues. The oxidative stress-related DEGs were marked in the volcano plots. (B) Bar plots show the oxidative stress-related DEGs between non-tumor and HCC tissues in each cell cluster.

Supplementary Figure 8. The ROC analysis of six gene signatures (GPX4, HMOX1, PRDX1, FOS, PRDX5, and TXN) for predicting the HCC early occurrence in external validation cohorts (GSE76427, GSE54236, GSE36376, GSE69715, GSE121248, GSE107170, and GSE45267) and the training cohort.

Supplementary Figure 9. The immunohistochemical pathological sections difference of six gene signatures (GPX4, HMOX1, PRDX1, FOS, PRDX5, and TXN) between the normal liver sample and HCC sample in the HPA (The Human Protein Atlas, https://www.proteinatlas.org/) database.

Supplementary Figure 10. KM analysis for progress-free interval and disease-specific survival status of low- and high-PRDX1 groups in TCGA cohort.

Supplementary Figure 11. Gene set enrichment analysis for PRDX1. (A, B) The biological process that PRDX1 is mainly involved in HCC patients. (C) In HCC patients, the activation or suppressed statue of PRDX1-related pathways.

Supplementary Figure 12. Immune infiltration differences in different PRDX1 expression groups.
